# Development of a tool for assessing the performance of long-term care systems in relation to care transition: Transitional Care Assessment Tool in Long-Term Care (TCAT-LTC)

**DOI:** 10.1186/s12877-023-04467-z

**Published:** 2023-11-20

**Authors:** Estera Wieczorek, Ewa Kocot, Silvia Evers, Christoph Sowada, Milena Pavlova

**Affiliations:** 1 Department of Health Economics and Social Security, Institute of Public Health, Faculty of Health Sciences, Jagiellonian University Collegium Medicum, Skawińska 8, 31-066, Krakow, Poland; 2 Department of Health Services Research, Care and Public Health Research Institute (CAPHRI), Maastricht University Medical Center, Faculty of Health, Medicine and Life Sciences, Maastricht University, P.O. Box 616, Maastricht, MD 6200 The Netherlands; 3 Faculty of Health, Medicine and Life Sciences, Maastricht University, P.O. Box 616, Maastricht, MD 6200 The Netherlands

**Keywords:** Integrated care, Coordinated care, Transitional care, Care transition, Patient-centered care, System performance, Long-term care, Organization, Financing

## Abstract

**Background:**

Improving the quality and safety of care transitions is a priority in many countries. Carrying out performance measurements play a significant role in improving quality of decisions undertaken by different actors involved in reforms. Therefore, the main objective of this paper is to present the development of an evaluation tool for assessing the performance of long-term care systems in relation to care transition, namely the Transitional Care Assessment Tool in Long-Term Care (TCAT-LTC). This study is performed as part of a larger European TRANS-SENIOR project.

**Methods:**

The development of the TCAT-LTC involved three steps. First, we developed a conceptual model based on Donabedian’s quality framework and literature review. Second, we carried out a thorough process of item pool generation using deductive (systematic literature review) and deductive-inductive methods (in-depth interviews) with experts in the field of long-term care. Third, we conducted preliminary validation of the tool by asking experts in research and practice to provide an opinion on a tool and to assess content validity. Future fourth step will involve a tool’s pilot with country experts from Germany, the Netherlands and Poland.

**Results:**

By applying methodological triangulation, we developed the TCAT-LTC, which consists of 2 themes, 12 categories and 63 items. Themes include organizational and financial aspects. Organizational aspects include categories such as communication, transfer of information, availability and coordination of resources, training and education of staff, education/support of the patient/informal caregiver, involvement of the patient/informal caregiver, telemedicine and e-Health, and social care. Financial aspects include categories such as primary care, hospital, and long-term care. We also present the instructions on the application of the TCAT-LTC.

**Conclusions:**

In this paper, we presented the development of the TCAT-LTC evaluation tool for assessing the performance of long-term care systems in relation to care transition. The TCAT-LTC is the first tool to assess the performance of long-term care systems in relation to care transition. Assessments can be carried out at the national and international level and enable to monitor, evaluate, and compare performance of the long-term care systems in relation to care transition within and across countries.

**Supplementary Information:**

The online version contains supplementary material available at 10.1186/s12877-023-04467-z.

## Background

Care transitions are vulnerable exchange points for older adults with complex care needs [1, 2]. Older persons often require care services from different practitioners in multiple settings, but practitioners tend to work in silos and are unaware of services delivered in previous settings [3]. Lack of coordination, communication, and transfer of information between the settings may lead to poorly executed transitions [4, 5]. Nonetheless, not only organizational aspects may affect the care transition of older adults. A recent study by Wieczorek and colleagues [6] pinpointed the importance of financial aspects (provider payment mechanism, reward, and penalty) and their impact on care transition in long-term care systems. A growing body of evidence suggests that a high proportion of care transitions among older adults is far from optimal. Fragmented care transitions are often associated with preventable adverse events, rehospitalizations and compromised patient outcomes [7–9]. Moreover, suboptimal care transitions may lead to unnecessarily high rates of health service use and health care spending in both, health and social care systems [10]. The recommendation of the World Health Organization is to avoid, if possible, or to optimize transitions between the settings as they are high-risk scenarios for patient safety [11]. Given the importance of this issue, improving the quality and safety of care transitions is an international priority, and efforts are being made by governments worldwide to optimize care transitions [12, 13].

Nonetheless, to improve quality of decisions undertaken by different actors such as practitioners, managers, governments, policymakers, and payers/insurers, health system performance measurements are needed [14]. Performance measurement instruments have two important goals, first, to promote accountability, and second, to improve the performance of the system. According to Donabedian [15], there are three approaches to assessment. The first approach focuses on the “structure”; the second one focuses on the “process” and the last one on “outcomes”. Assessments examining the “structure” study the settings and instrumentalities with which care is delivered. It might refer to the adequacy of facilities and equipment but also to the training and qualifications of the staff. At the same time, examining “process of care” allows us to answer the question: of whether health care (in this case, transitional care) is properly practised. Process measures may be indicators of future success or failure [15]. Process indicators are easy to measure, to interpret, provide clear pathways for action, and capture aspects of care that are valued by patients [16]. The last approach focuses on “outcomes” and has been widely used as an indicator of the quality of medical care. Outcome indicators reflect the impact of the health care service on the patient. Examples of outcome measures include mortality, survival, disease prevalence etc. Nevertheless, the use of outcome as the criterion for quality is questioned because many other factors other than medical care could affect the outcome [16].

Currently, to the best of our knowledge, there is no assessment tool dedicated to measuring the performance of long-term care systems in relation to care transition. Existing tools, such as Care Transition Measure (CTM) and Partners at Care Transitions Measure (PACT-M) do not assess care transition as part of the long-term care system. There are plenty of measures that assess only selected aspects related to care transition (e.g., discharge planning, patients’ experience) or focus on care transition between specific settings such as the hospital, home etc. [17–19]. For instance, the Care Transition Measure (CTM) is a tool used to assess the quality of the transition between hospital and home [20]. Similarly, PACT-M also focuses on care transition from hospital to home [21]. Existing tools, even though valued, have a narrow focus. According to the Institute of Medicine [22] and the report “To Err is Human” efforts to improve patient safety should be centered around the system rather than providers. Likewise, OECD report titled “Caring for Quality in Health” also emphasizes the importance of systemic changes and their impact on quality and efficiency of care [23]. For the purpose of this study, we define long-term care system as all organizations, providers, individuals, and actions with the primary aim to promote, maintain and/or improve the wellbeing, health and functional ability of individuals with limitations in intrinsic capacity [24].

The main objective of this paper is to present the development of an evaluation tool for assessing the performance of long-term care systems in relation to care transition. We provide details of the methods used to develop this tool, which was named Transitional Care Assessment Tool in Long-Term Care (TCAT-LTC), as well as the tool itself and the guide on how to apply it. The results of the application of the tool will be reported elsewhere. This study is performed as part of a larger European TRANS-SENIOR project focused on avoiding unnecessary care transitions and improving care for transitions that are needed.

## Methods

**Fig. 1:**
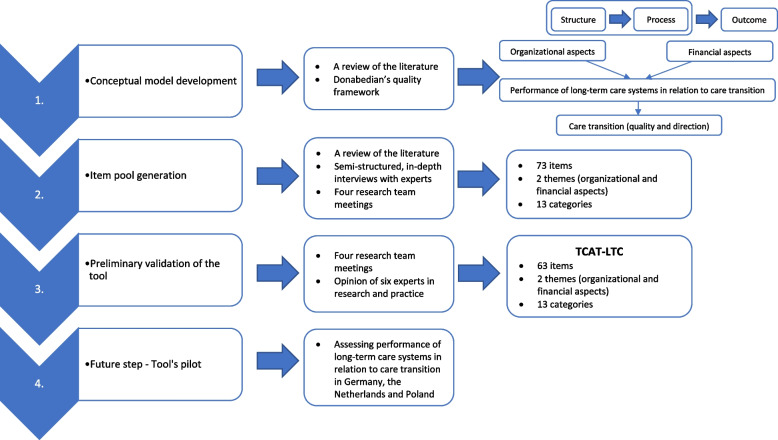
Flowchart of the tool development process

The development of the TCAT-LTC involved three steps (Fig. 1). We followed guidelines on scale development by DeVellis [25]. First (1), we developed a conceptual model based on Donabedian’s quality framework and literature review carried out by Wieczorek and colleagues [4, 6, 15]. Second (2), we carried out a thorough process of item pool generation using deductive and inductive methods as recommended by DeVellis and Morgado [25, 26]. In this step, we performed a systematic literature review (deductive method) and semi-structured, in-depth interviews (deductive-inductive method) with experts in the field of long-term care. Third (3), we conducted preliminary validation of the tool by asking experts in research and practice to provide an opinion on the tool and to assess content validity. Future fourth step will involve a tool’s pilot with country experts from Germany, the Netherlands and Poland.

### Step 1. Development of a conceptual model

For the purpose of this study, we defined transitional care as “a set of actions designed to ensure the coordination and continuity of health care as patients transfer between different locations or different levels of care within the same location. Representative locations include (but are not limited to) hospitals, sub-acute and post-acute nursing facilities, the patient’s home, primary and specialty care offices, and long-term care facilities” [27] p556. Thus, in this study, we focus on care transitions occurring in both, health care and social care sector, and between those sectors. We adopt this approach given the focus of our study on long-term care systems. World Health Organization [24] suggests that a long-term care system encompasses all organizations, providers, individuals, and actions that’s objective is to promote, maintain or improve the wellbeing, health, and functional ability of persons with limitations in intrinsic capacity. Moreover, given that the presented study is conducted along the European TRANS-SENIOR project that focuses on the optimization of care transitions of older adults, the primary focus of this study is on older adults. This patient group is particularly often in need of long-term care services and therefore, at higher risk of care transitions. Even though, the focus of our study is on older adults, the results of this study could be used for other patient groups as well. However, it is crucial to consider the specific needs of studied groups that might differ from those of older patients. We built the assessment tool involving two approaches out of three proposed by Donabedian, namely structure and process [15]. By focusing on these two approaches, we want to provide the evaluators with a better understanding of the relative magnitude of associations between structure and process and their impact on quality of care [15]. Through a literature review, we defined important core organizational and financial aspects that are relevant to care transition and decided that TCAT-LTC will focus on the following areas:

- How well is long-term care system performing when it comes to organizational aspects of care transition?
- How well is long-term care system performing when it comes to financial aspects of care transition?

### Step 2. Item pool generation

Item pool generation had two phases. First, we used a combination of deductive and inductive methods to build on the item pool, namely, we conducted a systematic literature review and semi-structured in-depth interviews with experts in long-term care. Second, we carried out multiple meetings with the research team to discuss the relevance and clarity of items and to refine the item list.

### Literature review

We used MEDLINE, Embase and CINAHL to search for relevant studies between 2005 and 2020 using three components to build the search terms: (1) old or geriatric or senior; (2) care transition or coordinated care or care continuity; (3) financing or organization. The search strategy was consulted with an academic health sciences librarian. The detail on the review methodology can be found in the published articles [4, 6] and on the International Prospective Register of Systematic Reviews (PROSPERO) platform under identification number CRD42020162566. The review results were used to build on the item pool by identifying key core organizational and financial aspects that are relevant for care transition.

### Semi-structured, in-depth interviews with experts

#### Design

We used a qualitative research design to understand what kind of organizational and financial aspects affect care transition in long-term care systems. Detailed information on the interviews is provided in Appendix 1 using the COnsolidated criteria for REporting Qualitative research (COREQ) checklist [28]. Below, some key methodology aspects are presented.

#### Participants

We used a purposive sampling method to identify country experts in long-term care and care transition in Germany, the Netherlands, and Poland. To be included in the study, participants had to (1) represent either providers from primary care, hospital, long-term care or payers/insurers. Also, they had to (2) have some experience with care transitions of older adults and (3) be familiar with one of the long-term care systems in Germany or the Netherlands or Poland. They also had to (4) speak English, German or Polish. We contacted by e-mail 23 potential participants and only one of the approached participants did not respond to the invitation to the study. We provided the respondents with detailed information about the study prior to the interview. All participants suggested the time and the mode/place for the interview. All the interviewees provided informed consent and voluntarily participated in the study. In total, 22 semi-structured interviews were conducted with country experts (8 experts from Germany, 8 experts from the Netherlands (one dyadic interview) and 7 experts from Poland).

#### Data collection

Interviews were conducted by the main researcher Estera Wieczorek (EW) with the help of a second researcher Christoph Sowada (CS). More information about the members of the research team and interviews can be found in the Appendix 1. At first, the interview guide was built based on the results from the literature review. The interview guide was discussed, modified, and accepted by the research team. The relevant topic list can be found in the Appendix 2. The first three interviews confirmed that the guide was clear to participants and thus, no adjustments were needed. The interviews were scheduled in the place/mode and at the time suggested by the participant. Majority of the interviews (18 out of 22) were carried out online due to the COVID-19 pandemic. Three interviews were face-to-face and carried out in the workplace of the participants, and one respondent provided the answers through e-mail. All interviewees were carried out once (without repeated interviews) with only the participant and an interviewer/s being present. Each interview lasted, on average 52 minutes (range: 27-107 minutes) and was recorded. Field notes were also taken during the interview. We then transcribed the recordings using Verbatim method (word by word) and sent the transcripts for a member check. Only 2 respondents provided some minor changes to the transcripts. Ethical considerations regarding this study are explained in the Appendix 1.

#### Data analysis

All the data was downloaded, coded, and analyzed using the method of qualitative content analysis. The analysis was facilitated with the use of ATLAS.ti Version 22. All interviews were coded using a deductive-inductive approach, i.e. the initial set of codes (themes/categories) was informed by the priori literature review, while additional codes (sub-themes/sub-categories) emerged from the interviews. Interviews in English and Polish were coded by the main researcher EW, who is a native Polish speaker, and a fluent English speaker. Interviews in German were coded by a second researcher CS, who is a native German speaker, fluent Polish, and English speaker; the main researcher EW was also involved to ensure uniformity of coded data. The results were used to challenge the categories coming from the literature review, refine categories, and develop items.

### Step 3. Preliminary validation of the tool

The preliminary validation of the tool was performed in two stages. First, the tool was discussed at four separate research team meetings to check for the clarity of the items and to agree on the first draft of the final item pool. Second, we sent an invitation by e-mail to 6 experts in research and practice to preliminary validate the TCAT-LTC tool. Expert panel consisted of 5 experts - two professors and an associate professor in aging and long-term care, an associate professor and assistant professor in health system organization and financing. Experts received an online document and were requested to fill out the form regarding TCAT-LTC tool. The form included a definition of transitional care and short information about the study, the questions regarding the relevance and clarity of each indicators/items. Relevance of an item was rated using a rating scale with 3 response categories: “very relevant”, “somehow relevant”, “not relevant”. Moreover, next to each indicator, experts were invited to provide comments and suggestions for improvement. At last, the form included optional fields where experts could provide general comments and suggestions regarding each category of indicators (e.g., communication), and propose items that should be added to each category. Respondents had 4 working days to provide responses and to send the filled form back by e-mail. All experts could contact the main researcher EW in case of questions. After receiving responses from the experts, the research team met again to analyze the responses. The results were used to review and refine items and categories, and to further improve the tool.

## Results

### Step 1. Development of conceptual model

**Fig. 2:**
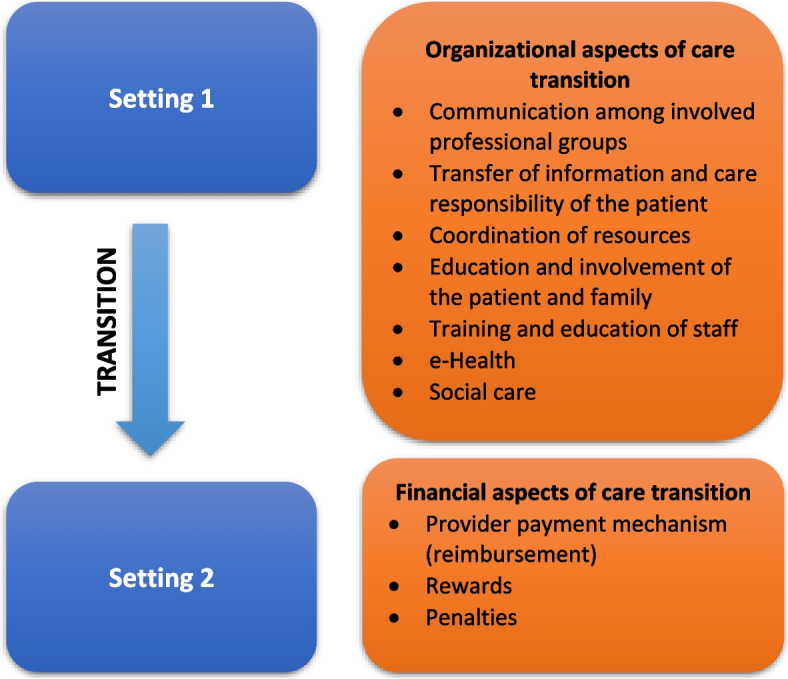
Organizational and financial aspects that affect care transition

Based on Donabedian’s three-components approach, structure measures may have an effect on process measures, and ultimately affect the outcome measures [15]. Based on Donabedian’s quality framework, organizational and financial aspects could be recognized as structure and process indicators. Systematic literature review that we performed served as a theoretical foundation and was conducted to identify general organizational and financial aspects that may affect care transition (Fig. 2). In line with Donabedian’s model, these aspects may affect the outcome (e.g. quality of care transition).

### Step 2. Item pool generation

The literature identified in the search pointed out to multiple organizational and financial aspects that may affect care transition in long-term care systems. Organizational aspects included: communication among involved professional groups, transfer of information and care responsibility of the patient, coordination of resources, education and involvement of the patient and family, training and education of staff, e-Health and social care. Financial aspects included: provider payment mechanism, rewards and penalties. More detailed information on the review findings can be found in recently published articles [4, 6]. Findings from the systematic review provided us with a guiding framework for developing the qualitative study.

After developing the guiding framework for our qualitative study, we conducted 22 interviews with country experts from Germany, the Netherlands and Poland (8 experts from Germany, 7 experts from the Netherlands and 7 experts from Poland). Of those, 18 participants represented providers (7 individuals represented long-term care, 6 primary care and 5 hospital), and four respondents represented payers/insurers. The analysis of the in-depth interviews revealed important organizational and financial aspects affecting care transition in their countries. The exact results and codes for each category, for each country can be found in Appendix 3. We used the responses from the experts to challenge the categories coming from the literature review, refine categories, and develop items. During interviews, experts were asked to discuss in detail all organizational and financial aspects that may affect care transition. There were also requested to indicate potential problems and solutions. Their responses enabled us to build a detailed and comprehensive item pool by developing items for each category. For instance, when discussing the category related to availability and coordination of resources, experts suggested a different type of resources relevant for care transition, among others – human resources. Moreover, country experts elaborated on communication in more detail and provided us with items that make communication effective (e.g., timely and direct communication between providers). At the same time, we also used responses from the interview to create new categories of items. For example, some respondents emphasized the importance of including patient and carer in decision-making process and considering their preferences. As a result, involvement of the patient/family/informal caregivers’ category was added.

### Step 3. Preliminary validation of the tool

Research team members met 4 times to analyze and refine each category and item included in the tool. After each session, adjustments to the tool have been made by unanimous decision of the team members. During the fourth meeting, the research team agreed on the final version of the tool, which was sent to six experts for validation. All six experts in research and practice responded to our invitation to provide us with their opinion and feedback on the tool. Nonetheless, one of the experts could not provide the response due to time constraints. Five of the experts sent their responses via e-mail and provided us with the items’ relevance rating, comments, and suggestions for improvement. Almost all experts recognized the relevance of the items included. Nevertheless, for a couple of items, the relevance and clarity were questioned. Experts also proposed to clarify and merge some items. After receiving filled forms from the experts, the research team met again to analyze each response. As a result, we adjusted the names of categories, combined, or removed items following the sumscore decision rule (defined as the total score for an item across all judges) (threshold accepted - more than 50% of respondents had to consider the item as “not relevant”) [26], and we changed the names of some items. The results on the relevance of each item can be found in Appendix 4. Additionally, we added some more explanations to some items. During an online meeting research team unanimously agreed on the new version of the tool.

### Transitional Care Assessment Tool in Long-Term Care (TCAT-LTC)

By applying methodological triangulation based on the three steps presented above, we finalized the TCAT-LTC presented in Table 1. The tool focuses on care transitions occurring in both, health care and social care sector, and between those sectors. TCAT-LTC is designed as an assessment tool that can be used internally or externally by different stakeholders at different levels of the LTC system. TCAT-LTC consists of 2 themes, namely, organizational and financial aspects. Organizational aspects are divided into 8 categories, and there are 3 categories regarding financial aspects. Organizational aspects include categories: communication, transfer of information, availability and coordination of resources, training and education of staff, education/support of the patient/informal caregiver, involvement of the patient/informal caregiver, telemedicine and e-Health, social care. Financial aspects include following categories: primary care, hospital, long-term care. Each category entails dedicated items. In total, TCAT-LTC consists of 63 items. TCAT-LTC could be completed by hand or electronically. Optimally, the assessment should be carried out by at least 2 experts in the field of transitional care of older adults. Moreover, the experts should be aware of the functioning and financing of health and long-term care systems in the assessed country. Experts might make use of data previously collected for other reports and assessments, for instance, health system performance assessment framework of a given country. Nonetheless, some of the information will need to be generated anew. With all necessary information available, the completion of the assessment takes around 2-3 hours, depending on the level of expertise of evaluators. We recommend performing an assessment of the performance of long-term care systems in relation to care transition using TCAT-LTC at least once a year.

**Table 1:** Transitional Care Assessment Tool in Long-Term Care (TCAT-LTC)

**Category/sub-category**	**Indicator**	**Country 1/** **Region 1**	**Country 2/ Region 2**	**Country 3/ Region 3**
**Organizational aspects**				
**1. Communication**	1.1 The use of interprofessional meetings within one setting in specific complex cases			
	1.2 Direct communication between different providers			
	1.3 On time communication			
	1.4 Communication of providers and other health and social institutions (if needed)			
	1.5 Communication of 3 sides (sending-patient/ informal caregiver-receiving)			
**2.Transfer of information**	2.1 Standardized/structured discharge information			
	2.2 Completeness of transferred information			
	2.3 Timeliness of transferred information			
	2.4 Responsibility for transferring information			
	2.5 Transferring information regarding patients’ and/or informal caregivers’ preferences			
**3. Availability & Coordination of resources**	3.1 Number of beds in LTC facilities			
	3.2 Number of staff in LTC			
	3.3 Waiting time for LTC			
	3.4 Number of social care workers			
	3.5 Regular meetings of involved providers/institutions			
	3.6 Availability and involvement of care coordinator			
	3.7 Coordinated discharge process by sending - receiving party			
	3.8 Access to physiotherapists/rehabilitation			
	3.9 Involvement of primary care			
	3.10 Assessing informal caregivers’ ability to provide appropriate care (if applicable)			
**4. Training and education of staff**	4.1 Availability of trainings regarding transitional care			
	4.2 Availability of trainings provided to case managers/care coordinators (if applicable)			
	4.3 Availability of trainings for care assistants (if applicable)			
	4.4 Obligation to uptake additional courses/trainings			
**5. Education/support of the patient/informal caregivers**	5.1 Access to education/advise/information for patient and/or informal caregivers (related mostly to medical & caring needs)			
	5.2 Access to information (related to administrative/organizational aspects)			
	5.3 Reimbursement of trainings/courses for informal caregivers			
	5.4 Access to coordinator guiding through the transition process			
	5.5 Access to instrumental support			
	5.6 Access to respite care services			
	5.7 Financial renumeration of informal caregivers			
**6. Involvement of the patient/informal caregiver**	6.1 Involving patient & informal caregiver in decision-making process			
	6.2 Considering patients’ expressed preferences, if possible			
	6.3 Considering informal caregivers’ expressed preferences, if possible			
**7. Telemedicine and e-Health**	7.1 Access to electronic patient record			
	7.2 The use of medical technologies, e-Health to monitor patients’ health			
	7.3 Availability of telephone consultations			
	7.4 Availability of video consultations			
	7.5 Access to tele-information			
**8. Social care**	8.1 Involvement of social care workers to look after the patient			
	8.2 Social care worker involvement in discharge process (in hospital)			
	8.3 Social care worker prepares patient & informal caregiver			
	8.4 Social care worker prepares receiving setting			
	8.5 Social care worker competencies and responsibilities			
**Financial aspects**				
**9. Primary care**	9.1 Appropriateness of reimbursement level - sufficient reimbursement level to cover the costs?			
	9.2 Presence of incentives that stimulate cost-efficient care			
	9.3 Sufficient renumeration level of the staff			
	9.4 Compensation for care coordinator/coordination			
	9.5 Reimbursement for transitional care			
	9.6 Out-of-pocket payments			
**10. Hospital**	10.1 Appropriateness of reimbursement level - sufficient reimbursement level to cover the costs?			
	10.2 Presence of incentives that stimulate cost-efficient care			
	10.3 Sufficient renumeration level of the staff			
	10.4 Compensation for care coordinator/coordination			
	10.5 Reimbursement for transitional care			
	10.6 Out-of-pocket payments			
**11. Long-term care**	11.1 Appropriateness of reimbursement level - sufficient reimbursement level to cover the costs?			
	11.2 Presence of incentives that stimulate cost-efficient care			
	11.3 Sufficient renumeration level of the staff			
	11.4 Compensation for care coordinator/coordination			
	11.5 Reimbursement for transitional care			
	11.6 Out-of-pocket payments			
	11.7 Financial contribution by social care institutions to cover LTC costs			

Each question/item can be graded on a three-grade scale. Depending on the answer, countries can score 3, 2 or 1 points, where 3 points are the highest score, and 1 point is the lowest score. If the answer for an item was “not applicable” then the item is excluded from the assessment. Similarly, in case of missing data, there should be an annotation “missing data”, and such an item is excluded from the assessment. Nonetheless, respondents may use “not applicable” and “missing data” options only in justified cases. The exact instructions for the scoring of each question in the TCAT-LTC can be found in Table 2.

**Table 2:** Transitional Care Assessment Tool in Long-Term Care (TCAT-LTC) Instruction

**Category/sub-category**		**Indicator**	**Explanation**	**Score 3**	**Score 2**	**Score 1**
**Organizational aspects**						
**1. Communication**		1.1 The use of interprofessional meetings within one setting in specific complex cases	Are different professionals from one setting meeting (e.g., in the form of round-table meetings) to discuss patients’ case in specific complex cases?	Yes, it is performed with nearly all complex cases	It is rarely performed, even in complex cases	No, it is almost never performed
		1.2 Direct communication between different providers	Are providers in personal contact regarding planned/during care transition? (e.g., hospital – primary care, long-term care – hospital, long-term care - social care, ambulatory long-term care – stationary long-term care). Consider all forms of communication (e.g., verbal, written, digital)	Yes, it is performed with nearly all patients	It is rarely performed, only in specific cases	No, it is almost never performed
		1.3 On time communication	Is communication between providers on time and without delay?	Yes, communication between providers is on time	Communication is often delayed and affect continuity of care	Communication is always delayed and affect continuity of care
		1.4 Communication of providers and other health and social institutions (if needed)	Is there communication between providers and other health and social institutions (including payers/insurers/organizers of long-term care) regarding patient’s case?	Yes, it is performed with nearly all patients	It is rarely performed, only in specific cases	No, it is almost never performed
		1.5 Communication of 3 sides (sending-patient/ informal caregiver-receiving)	Is patient and/or informal caregiver involved in communication between sending and receiving setting?	Yes, it is performed with nearly all patients	It is rarely performed, only in specific cases	No, it is almost never performed
**2.Transfer of information**		2.1 Standardized/structured discharge information	Is discharge information standardized/structured?	Yes, there is standardized/structured discharge information provided for nearly all patients	Standardized/structured discharge information is rarely provided	No, standardized/structured discharge information is never performed
		2.2 Completeness of transferred information	Is transferred information complete and includes all essential information to provide high-quality care?	Yes, transferred information is always complete and includes all essential information	Transferred information is often incomplete and doesn’t include all essential information	No, transferred information is always incomplete and doesn’t include all essential information
		2.3 Timeliness of transferred information	Is transfer of information on time?	Yes, information is almost always transferred on time	Transferred information is rarely on time	No, transferred information is always delayed and never on time
		2.4 Responsibility for transferring information	Is there an individual responsible for transferring information? Is there a person who can be contacted in case of any issue?	Yes, there is a person responsible for transferring information that can be contacted in case of any issue	There is a person responsible for transferring information, but there is no one who could be contacted in case of any issue	No, there is no person responsible for transferring information and there is no person who could be contacted in case of any issue
		2.5 Transferring information regarding patients’ and/or informal caregivers’ preferences	Are patients’ and/or informal caregiver preferences included in the transferred information? (e.g., preferences concerning long-term care placement, medical treatment, activities of daily life living)	Yes, patients’ and/or informal caregivers’ preferences are always included in the transferred information	Patients’ and/or informal caregivers’ preferences are rarely included in the transferred information	No, patients’ and/or informal caregivers’ preferences are never included in the transferred information
**3. Availability & Coordination of resources**		3.1 Number of beds in LTC facilities	Is number of beds in different LTC facilities sufficient to address older population needs?	Yes, the number of beds in different LTC facilities is sufficient to address older population needs	The number of beds in different LTC facilitates is limited/insufficient	No, the number of beds in different LTC facilities is very limited and very far from addressing older population needs
		3.2 Number of staff in LTC	Is number of staff sufficient in LTC to address older population needs?	Yes, the number of staff in LTC is sufficient to address older population needs	The number of staff in LTC is limited/insufficient to address older population needs	No, the number of staff in LTC is very limited and very far from addressing older population needs
		3.3 Waiting time for LTC	Are patients able to access LTC without waiting time?	Yes, nearly all patients can access LTC without any waiting time	Patients often experience long waiting time to access LTC	No, nearly all patients experience long waiting time to access LTC
		3.4 Number of social care workers	Is number of social care workers sufficient to address older population needs?	Yes, the number of social care workers is sufficient to address older population needs	The number of social care workers is limited/insufficient to address older population needs	No, the number of social care workers is very limited and very far from addressing older population needs
		3.5 Regular meetings of involved providers/institutions	Are there regular meetings of stakeholders - including all providers from health care and social system, organizers, payers, involved in the transition process?	Yes, there are regular meetings with different stakeholders	Meetings with different stakeholders are rarely performed	No, meetings with different stakeholders are almost never performed
		3.6 Availability and involvement of care coordinator	Is there a care coordinator available? Is care coordinator actively involved especially in complex cases?	Yes, there is a care coordinator, and he/she is actively involved in nearly all complex cases	There is no care coordinator but someone else performs a similar function, but he/she is rarely actively involved in complex cases	No, there is no care coordinator
		3.7 Coordinated discharge process by sending - receiving party	Is discharge process coordinated between sending – receiving party?	Yes, the entire discharge process is coordinated between sending – receiving party for nearly all patients	The discharge process is rarely coordinated between sending - receiving party	No, the discharge process is almost never coordinated between sending – receiving party for any patient
		3.8 Access to physiotherapists/rehabilitation	Is there an access to physiotherapists/rehabilitation in a setting, for instance, primary care, hospital, long-term care?	Yes, nearly all patients can access physiotherapists/rehabilitation without any problem	Patients rarely have access to physiotherapists/rehabilitation	No, nearly all patients do not have access to physiotherapists/rehabilitation
		3.9 Involvement of primary care	Is primary care involved in patients’ care at all levels/in all settings?	Yes, primary care is involved in nearly all patients care at all levels/in all settings	Primary care is rarely involved in patients’ care at all levels/in all settings, only in specific cases	No, primary care is almost never involved in patients’ care at all levels/in all settings
		3.10 Assessing informal caregivers’ ability to provide appropriate care (if applicable)	Is informal caregivers’ ability to provide appropriate care required at home assessed?	Yes, it is performed with nearly all informal caregivers	It is rarely performed, only in specific cases	No, it is almost never performed
**4. Training and education of staff**		4.1 Availability of trainings regarding transitional care	Are there trainings regarding general aspects of transitional care available for all the staff included in transitional care?	Yes, trainings are available	Trainings are rarely available	No, there is no trainings for the staff
		4.2 Availability of trainings provided to case managers/care coordinators (if applicable)	Are there specialized trainings for case managers/care coordinators available?	Yes, trainings are available	Trainings are rarely available	No, there is no trainings for the staff
		4.3 Availability of trainings for care assistants (if applicable)	Are there trainings on how to provide care to older patient available for care assistants?	Yes, trainings are available	Trainings are rarely available	No, there is no trainings for the staff
		4.4 Obligation to uptake additional courses/trainings	Is there an obligation for staff to uptake additional courses/trainings regarding transitional care?	Yes, there is an obligation	There is no obligation, but it is common for staff to uptake additional courses/trainings	No, there is no obligation
**5. Education/support of the patient/informal caregivers**		5.1 Access to education/advise/information for patient and/or informal caregivers (related mostly to medical & caring needs)	Is there availability of places to educate/advise/inform patient and/or informal caregivers? Consider education/advise/information relating mostly to medical & caring needs.	Yes, places to educate/advise/inform are widely available to nearly all patients and/or informal caregivers	Places to educate/advise/inform are rarely available to patients and/or informal caregivers	No, there is almost no places that educate/advise/inform patients and/or informal caregivers
		5.2 Access to information (related to administrative/organizational aspects)	Is there availability to essential information (including administrative/organizational information) provided to the patient and/or informal caregivers?	Yes, essential information (including administrative/organizational information) is widely available it is provided to nearly all patients and/or informal caregivers	It is rarely available, and it is rarely provided to patients and/or informal caregivers, only in specific cases	No, it is almost never available and provided to patients and/or informal caregivers
		5.3 Reimbursement of trainings/courses for informal caregivers	Are free trainings/courses for informal caregivers funded?	Yes, they are always funded	Trainings/courses for caregivers are funded only from time to time	No, trainings/courses are not funded
		5.4 Access to coordinator guiding through the transition process	Do patient and/or informal caregiver have access to coordinator that guide them through the transition process?	Yes, nearly all patients and/or informal caregivers have access to coordinator	Patients and/or informal caregivers rarely have access to coordinator	No, patients and/or informal caregivers almost never have access to coordinator
		5.5 Access to instrumental support	Do patient/informal caregiver have access to instrumental support (e.g. in form of material goods – wheelchairs, adjustable beds, services or task assistance) or at least support in finding these resources?	Yes, there is easy access to instrumental support to nearly all patients and/or informal caregivers	Access to instrumental support is limited, available only for some patients and/or informal caregivers but support in findings these resources is provided	No, nearly all patients and/or informal caregivers don’t have access to instrumental support
		5.6 Access to respite care services	Is there an access to respite care services for informal caregivers?	Yes, there is an access to respite care services	There is an access, but it is rarely used	No, there is no access to respite care services
		5.7 Financial renumeration of informal caregivers	Are informal caregivers financially compensated for providing care?	Yes, informal caregivers receive financial compensation	Financial compensation is received by informal caregivers only in specific cases	No, informal caregivers do not receive financial compensation
**6. Involvement of the patient/informal caregiver**		6.1 Involving patient & informal caregiver in decision-making process	Can patient & informal caregiver choose between solutions offered by the provider/institutions?	Yes, nearly all patients and/or informal caregivers can choose between solutions offered by the provider/institutions	Patients and/or informal caregivers rarely can choose between solutions offered by the provider/institutions	No, patients and/or informal caregivers can almost never choose between solutions offered by the provider/institutions
		6.2 Considering patients’ expressed preferences, if possible	Can patient express their preferences and are there any mechanisms that make providers liable to taken them into account? (e.g., financial incentives, legal obligations)	Yes, patients can express their preferences and there are mechanisms that make providers liable/encouraged to take them into account	Yes, patients can express their preferences, but there are no mechanisms that make providers liable/encouraged to take them into account	No, patients can’t express their preferences and there are no mechanisms that make providers liable/encouraged to take them into account
		6.3 Considering informal caregivers’ expressed preferences, if possible	Can informal caregivers express their preferences and are there any mechanisms that make providers liable/encouraged to taken them into account? (e.g., financial incentives, legal obligations)	Yes, informal caregivers can express their preferences and there are mechanisms that make providers liable/encouraged to take them into account	Yes, informal caregivers can express their preferences, but there are no mechanisms that make providers liable/encouraged to take them into account	No, informal caregivers can’t express their preferences and there are no mechanisms that make providers liable/encouraged to take them into account
**7. Telemedicine and e-Health**		7.1 Access to electronic patient record	Is there an access to electronic patient record in all settings?	Yes, there is an access to electronic patient record in all settings	There is an access to electronic patient record, but only in some settings	No, there is no access to electronic patient record in almost any setting
		7.2 The use of medical technologies, e-Health to monitor patients’ health	Are medical technologies used to monitor patients’ health?	Yes, it is performed with nearly all patients	It is rarely performed, only in specific cases	No, it is almost never performed
		7.3 Availability of telephone consultations	Are telephone consultations available to patients and/or informal caregivers? (refers to the medical consultation)	Yes, telephone consultations are available	Telephone consultations are rarely available, only in specific cases	No, telephone consultations are unavailable
		7.4 Availability of video consultations	Are video consultations available to patients and/or informal caregivers?	Yes, video consultations are available	Video consultations are rarely available, only in specific cases	No, video consultations are unavailable
		7.5 Access to tele-information	Do patients/informal caregivers have access to tele-information?	Yes, nearly all patients/informal caregivers have access to tele-information	Patients/informal caregivers rarely have access to tele-information	No, nearly all patients/informal caregivers do not have access to tele-information
**8. Social care**		8.1 Involvement of social care workers to look after the patient	Are social care workers actively involved to look after the patient at home?	Yes, social care workers actively look after the patient at home in nearly all patients’ cases	Social care workers rarely actively look after the patient at home	No, social care workers are almost never actively looking after the patient at home
		8.2 Social care worker involvement in discharge process (in hospital)	Are social care workers involved in discharge process? (In collaboration with health care worker)	Yes, social care workers are involved in nearly all discharge processes	Social care workers are rarely involved in discharge process	No, social care workers are almost never involved in any discharge process
		8.3 Social care worker prepares patient & informal caregiver	Does social care worker prepare patient & informal caregiver before the discharge? (In collaboration with health care worker)	Yes, social care worker prepares nearly all patients and informal caregivers before the discharge	Social care worker rarely prepares patients and informal caregivers, only in specific cases	No, social care worker almost never prepares patients and informal caregivers
		8.4 Social care worker prepares receiving setting	Does social care worker prepare receiving setting before the discharge? (In collaboration with health care worker)	Yes, social care worker prepares receiving setting for nearly all patients and informal caregivers	Social care worker rarely prepares receiving setting, only in specific cases	No, social care worker almost never prepares receiving setting
		8.5 Social care worker competencies and responsibilities	Do social care worker competencies and responsibilities allow for proactive engagement in care coordination?	Yes, social care workers have a lot of competencies and responsibilities that allow for proactive engagement in coordination	Social care workers have limited competencies and responsibilities	No, social care worker competencies and responsibilities are very limited and do not allow in proactive engagement in coordination
**Financial aspects**						
**9. Primary care**	9.1 Appropriateness of reimbursement level - sufficient reimbursement level to cover the costs?		Is the reimbursement level sufficient to cover the incurred costs?	Yes, the reimbursement level is sufficient to cover the costs incurred	The reimbursement level is lower than costs incurred but it does not have an impact on quality of care/services provided	No, the reimbursement level is lower than costs incurred, and it has an impact on quality of care/services provided
	9.2 Presence of incentives that stimulate cost-efficient care		Are there any incentives (e.g. Pay for Performance, Pay for Quality etc.) in place to stimulate cost-efficient care?	Yes, there are incentives in place	There are incentives, but only in some settings	No, there are no incentives in place
	9.3 Sufficient renumeration level of the staff		Is the renumeration for staff sufficient (according to the staff) to ensure the retention of staff?	Yes, the renumeration is sufficient	The renumeration is insufficient, but it has no impact on staff retention	No, the renumeration is insufficient and it has an impact on staff retention
	9.4 Compensation for care coordinator/coordination		Is there a compensation for care coordinator/coordination?	Yes, there is a compensation for care coordinator/coordination	There is a compensation for care coordination/coordination, but it is very low	No, there is no compensation for care coordinator/coordination
	9.5 Reimbursement for transitional care		Is there a reimbursement for transitional care?	Yes, there is a reimbursement for transitional care	There is a reimbursement for transitional care, but it is very low	No, there is no reimbursement for transitional care
	9.6 Out-of-pocket payments		Are out-of-pocket payments level low and do not affect patients & informal caregiver decision/possibility to access primary care?	Yes, the out-of-pocket payments are low and do not affect patients and informal caregiver decision/possibility to access primary care	Out-of-pocket payments are high but do not affect patients and informal caregiver decision/possibility to access primary care	No, the out-of-pocket payments are high and affect patients and informal caregiver decision/possibility to access primary care
**10. Hospital**	10.1 Appropriateness of reimbursement level - sufficient reimbursement level to cover the costs?		Is the reimbursement level sufficient to cover the incurred costs?	Yes, the reimbursement level is sufficient to cover the costs incurred	The reimbursement level is lower than costs incurred but it does not have an impact on quality of care/services provided	No, the reimbursement level is lower than costs incurred, and it has an impact on quality of care/services provided
	10.2 Presence of incentives that stimulate cost-efficient care		Are there any incentives (e.g. Pay for Performance, Pay for Quality etc.) in place to stimulate cost-efficient care?	Yes, there are incentives in place	There are incentives, but only in some settings	No, there are no incentives in place
	10.3 Sufficient renumeration level of the staff		Is the renumeration for staff sufficient (according to the staff) to ensure the retention of staff?	Yes, the renumeration is sufficient	The renumeration is insufficient, but it has no impact on staff retention	No, the renumeration is insufficient and it has an impact on staff retention
	10.4 Compensation for care coordinator/coordination		Is there a compensation for care coordinator/coordination?	Yes, there is a compensation for care coordinator/coordination	There is a compensation for care coordination/coordination, but it is very low	No, there is no compensation for care coordinator/coordination
	10.5 Reimbursement for transitional care		Is there a reimbursement for transitional care?	Yes, there is a reimbursement for transitional care	There is a reimbursement for transitional care, but it is very low	No, there is no reimbursement for transitional care
	10.6 Out-of-pocket payments		Are out-of-pocket payments level low and do not affect patients & informal caregiver decision/possibility to access hospital?	Yes, the out-of-pocket payments are low and do not affect patients and informal caregiver decision/possibility to access hospital	Out-of-pocket payments are high but do not affect patients and informal caregiver decision/possibility to access hospital	No, the out-of-pocket payments are high and affect patients and informal caregiver decision/possibility to access hospital
**11. Long-term care**	11.1 Appropriateness of reimbursement level - sufficient reimbursement level to cover the costs?		Is the reimbursement level sufficient to cover the incurred costs?	Yes, the reimbursement level is sufficient to cover the costs incurred	The reimbursement level is lower than costs incurred but it does not have an impact on quality of care/services provided	No, the reimbursement level is lower than costs incurred, and it has an impact on quality of care/services provided
	11.2 Presence of incentives that stimulate cost-efficient care		Are there any incentives (e.g. Pay for Performance, Pay for Quality etc.) in place to stimulate cost-efficient care?	Yes, there are incentives in place	There are incentives, but only in some settings	No, there are no incentives in place
	11.3 Sufficient renumeration level of the staff		Is the renumeration for staff sufficient (according to the staff) to ensure the retention of staff?	Yes, the renumeration is sufficient	The renumeration is insufficient, but it has no impact on staff retention	No, the renumeration is insufficient and it has an impact on staff retention
	11.4 Compensation for care coordinator/coordination		Is there a compensation for care coordinator/coordination?	Yes, there is a compensation for care coordinator/coordination	There is a compensation for care coordination/coordination, but it is very low	No, there is no compensation for care coordinator/coordination
	11.5 Reimbursement for transitional care		Is there a reimbursement for transitional care?	Yes, there is a reimbursement for transitional care	There is a reimbursement for transitional care, but it is very low	No, there is no reimbursement for transitional care
	11.6 Out-of-pocket payments		Are out-of-pocket payments level low and do not affect patients & c informal caregiver decision/possibility to access LTC?	Yes, the out-of-pocket payments are low and do not affect patients and informal caregiver decision/possibility to access LTC	Out-of-pocket payments are high but do not affect patients and informal caregiver decision/possibility to access LTC	No, the out-of-pocket payments are high and affect patients and informal caregiver decision/possibility to access LTC
	11.7 Financial contribution by social care institutions to cover LTC costs		Do social care institutions contribute to cover LTC costs for patients & informal caregiver that have financial problems to pay for LTC?	Yes, social care institutions help to cover LTC costs for nearly all patients and informal caregivers that need it	Social care institutions rarely cover LTC costs for patients and informal caregivers	No, social care institutions almost never cover LTC costs for patients and informal caregivers

^a^Some of the items in the TCAT-LTC tool might appear to be conceptual and aspirational. Therefore, they will need to be operationalized by the evaluators of a given LTC system considering the specificities of that LTC system and its context. Please see the changes in Table 2

At the end of the questionnaire, the total score can be calculated. Evaluators should first sum up the scores from all items for which responses were provided, and then divide the total sum by the maximum number of points that could be scored for all items (excluding items with answer “not applicable”, “missing data”). At last, the divided score should be multiplied by 100% to obtain score as a percentage.

For instance, a country scored 142 points in 61 items (2 items were excluded because there were not applicable), therefore, (142 / 183 * 100% = 77,6%). The score can be used as a rough indication on the performance of a country’s long-term care system in relation to care transition. The higher the percentage, the more items considered important for care transition have been addressed by the long-term care system.

## Discussion

The objective of this paper was to present the development of an evaluation tool for assessing the performance of long-term care systems in relation to care transition. We elaborated in detail on the methods used to develop the tool. The TCAT-LTC is, to our knowledge, the first tool that looks at the performance of long-term care systems in terms of organizational and financial aspects, and their relation to care transition.

The proposed TCAT-LTC assess long-term care performance in relation to care transition using a structure and process approach. The TCAT-LTC consists of 63 questions/items, grouped into 2 themes (organizational and financial) and 12 categories. Many of the items in the TCAT-LTC are related and may influence one another. For instance, the number of staff in LTC, number of beds in LTC facilities and appropriateness of reimbursement level may have an impact on waiting time for LTC. The TCAT-LTC shows the interrelation between organizational and financial aspects, and structure and process.

As confirmed by the experts’ validation, the TCAT-LTC is a helpful tool that separates the long-term care system into manageable parts by identifying organizational and financial aspects that are relevant to care transition. Assessments using the tool can be carried out at the national and international level to help to monitor, evaluate, and compare performance of the long-term care systems in relation to care transition within and across countries. Moreover, the TCAT-LTC aims to inform decision-makers and thus, improve the quality of the decisions undertaken by different stakeholders regarding care transition. Applying the TCAT-LTC enables us to shed light on high-performing countries when it comes to care transition in the long-term care systems. As a result, countries may use this knowledge to learn from pioneers by adapting strategies and solutions that proved to be effective.

Evaluation of long-term care (LTC) systems is very important but understudied subject. Monitoring the performance of long-term care systems is necessary for the identification of current issues and for informing evidence-based policy-making. Reforms cannot take place without a sound understanding of how long-term care system is performing. There are a few existing frameworks for LTC system performance assessment that originated in different parts of the world [29–32]. Their common goal is to better understand the LTC system. One of the tools measures Long-Term Services and Supports across five dimensions, including effective transitions. Nonetheless, this tool uses an outcome approach to performance instead of structure and process [30]. Such approach has certain limitations and should be used with discrimination as suggested by Donabedian [15].

We acknowledge that the completion of this tool might have the unintentional effect of diverting resources. Nevertheless, the completion of the tool by staff that is familiar with transitional care and LTC of older adults should not take longer than 2-3 hours. Performing assessment with the TCAT-LTC is an essential step in promoting accountability and improving the performance of the LTC system.

### Limitations

Although we performed an exhaustive process of tool development, this study has some limitations. First, we are aware that the literature review that we performed may not have identified all relevant literature due to heterogeneity of terminology for care transitions. Moreover, qualitative interviews were carried out by two interviewers and in three different languages. Therefore, there may have been some discrepancies between the interviewers and between the languages in which the interviews were carried out. Furthermore, for the theoretical analysis, we did not use target population opinion to theoretically refine the items and to analyze the tools’ content validity. Instead, we only used expert judges. Future studies are recommended to involve target population groups as it enables to identify and eliminate potential problems in the scale (to test the language and level of comprehension). Another limitation of our study is the absence of direct input from patients and their informal caregivers. We acknowledge that involving their opinion and perspectives is important in future research and policymaking. We are also aware that some of the items in the tool might not be specific enough, and this may cause an ambiguous understanding of the items. Few non-specific items in our tool are due to the variability and complexity of long-term care systems that could be assessed with this tool. Given, there is still a need for thorough validation of the tool. Future validation might further refine items that enable us to provide more detailed and clear explanations of the scoring system. Tools’ pilot test is our next step. We plan to test the TCAT-LTC in Germany, the Netherlands and Poland.

### Strengths

Our study had some strengths as well. Item generation process is one of the most important steps in the scale development process. For this purpose, we used a combination of both deductive and inductive approaches for item generation to strengthen the validity of the tool. Twenty-five different experts in the field of long-term care and transitional care from three different countries – Germany, the Netherlands and Poland were involved at different stages in this study. This comprehensive approach helped us to ensure that key items are included in the tool.

## Conclusions

In this paper, we presented the development of the TCAT-LTC evaluation tool for assessing the performance of long-term care systems in relation to care transition. We also presented the instructions on the application of the TCAT-LTC. The TCAT-LTC is the first tool to assess the performance of long-term care systems in relation to care transition. Assessments using the TCAT-LTC can be carried out at the national and international level, which can help to monitor, evaluate, and compare the performance of the long-term care systems (in relation to care transition) within and across different countries. Performing assessment with the TCAT-LTC can be an important first step toward optimizing care transitions for older adults and their informal caregivers. This is particularly important due to ageing population and thus, increased proportion of individuals with complex health and social care needs. Feedback on the application of the tool is welcomed as it will help us to further refine the TCAT-LTC.

### Supplementary Information

**Additional file 1: Appendix 1.** Qualitative study protocol.

**Additional file 2: Appendix 2.** Financial and organizational aspects of care transition in the long-term care systems.

**Additional file 3: Appendix 3.** Basic findings from the interviews.

**Additional file 4: Appendix 4.** Transtitional Care Assessment Tool (TCAT) – Relevance of the items.

## Data Availability

The datasets used and/or analyzed during the current study are available from the corresponding author on reasonable request.

## Abbreviations

COREQ: COnsolidated criteria for REporting Qualitative research
LTC: Long-Term Care
PACT-M: Partners at Care Transitions Measure
PROSPERO: International Prospective Register of Systematic Reviews
TCAT-LTC: Transitional Care Assessment Tool in Long-Term Care
CTM: Care Transition Measure
