# Supplementary material for: Development of a tool for assessing the performance of long-term care systems in relation to care transition: Transitional Care Assessment Tool in Long-Term Care (TCAT-LTC)
Source: BMC Geriatr. 2023 Nov 20;23:760. doi: 10.1186/s12877-023-04467-z (PMC10662551; doi:10.1186/s12877-023-04467-z)
Supplement: Supplementary file 1 — Additional file 1: Appendix 1. Qualitative study protocol. [file 12877_2023_4467_MOESM1_ESM.docx]

**Appendix 1**

**Qualitative study protocol**

**Semi-structured, in-depth interviews with experts**

We use COnsolidated criteria for REporting Qualitative research (COREQ) checklist to report on our qualitative research.

*Design*

We used a qualitative research design to understand what kind of organizational and financial aspect affect care transition in long-term care systems.

*Participants*

We used a purposive sampling method to identify country experts in long-term care and care transition in Germany, the Netherlands, and Poland. To be included in the study, participants had to (1) represent either providers from primary care, hospital, long-term care or payers/insurers. Also, they had to (2) have some experience with care transitions of older adults and (3) be familiar with one of the long-term care systems in Germany or the Netherlands or Poland. They also had to (4) speak English, German or Polish.

We contacted by e-mail 23 potential participants and only one of the approached participants did not respond to the invitation to the study. We provided the respondents with detailed information about the study prior to the interview. Only one of the approached participants did not respond to the invitation to the study. With all the other participants the time and the mode/place of the interview were agreed on. All the interviewees provided informed consent and voluntarily participated in the study. Finally, 22 semi-structured interviews were conducted with country experts (8 experts from Germany, 7 experts from the Netherlands and 7 experts from Poland).

*Data collection*

Interviews in English were conducted by the main researcher, Estera Wieczorek (EW) (MSc) (female), holding master’s degree in Healthcare Policy, Innovation and Management and master’s degree in Global Health. Interviews in German were conducted by another researcher, Christoph Sowada (CS) (Prof) (male) holding PhD in Economics. EW is a PhD candidate at the Jagiellonian University and Maastricht University while CS works at the department of Health Economics and Social Security in the Jagiellonian University Collegium Medicum in Poland. Prior to this study, EW and CS followed a course on how to conduct qualitative studies and had some practical experience in conducting interviews.

At first, the interview guide was built based on the results from the literature review. The interview guide was discussed, modified, and accepted by the research team. The first three interviews confirmed that the interview guide was clear to participants and thus, no adjustments were needed. The interviews were scheduled in the place/mode and at the time suggested by the participant. Majority of the interviews (18 out of 22) were carried out online due to the COVID-19 pandemic. Three interviews were face-to-face carried in the workplace of the participants, and one respondent provided the answers through e-mail.

All interviewees were carried out once (without repeated interviews) with only the participant and an interviewer/s being present. Each interview lasted approximately 45-60 minutes, and was recorded. The field notes were also taken during the interview.

We then transcribed the recordings using Verbatim method (word by word) and sent the transcripts for a member check. Only 2 respondents provided some minor changes to the transcripts.

*Ethical consideration*

Throughout our qualitative study, one of our main objectives was to provide all the participants with the respect, sensitivity, reduced risk of harm and exploitation. For this reason, we closely followed the principles outlined in the declaration of Helsinki. Our study was approved by the Ethical Committee at the Jagiellonian University (Poland) (approval number 1072.6120.54.2021) and Maastricht University (The Netherlands) (approval number FHML-REC/2021/079). The ethical approval for this study was not needed in Germany. This was confirmed by the Institute for Health Care and Nursing Studies at Martin-Luther University Halle-Wittenberg in Germany. Informed consent in a written form was provided by all participants prior to the interview.

*Data analysis*

All the data was downloaded, coded, and analyzed using the method of qualitative content analysis. The analysis was facilitated with the use of ATLAS.ti Version 22. All interviews were coded using a deductive-inductive approach, i.e. the initial set of codes (themes/categories) was informed by a priori literature review, while additional codes (sub-themes/sub-categories) emerged from the interviews. Interviews in English and Polish were coded by the main researcher E.W. (native Polish speaker, and fluent English speaker), while interviews in German were coded together by another researcher CS (native German speaker, fluent Polish, and English speaker) while the main researcher EW was also involved to ensure uniformity of coded data.
