# Supplementary material for: Development of a tool for assessing the performance of long-term care systems in relation to care transition: Transitional Care Assessment Tool in Long-Term Care (TCAT-LTC)
Source: BMC Geriatr. 2023 Nov 20;23:760. doi: 10.1186/s12877-023-04467-z (PMC10662551; doi:10.1186/s12877-023-04467-z)
Supplement: Supplementary file 2 — Additional file 2: Appendix 2. Financial and organizational aspects of care transition in the long-term care systems. [file 12877_2023_4467_MOESM2_ESM.docx]

**Appendix 2**

**Project Title: Financial and organizational aspects of care transition in the long-term care systems.**

**Topic list – Interview and questionnaire**

Investigator Name: Estera Wieczorek, Silvia Evers, Milena Pavlova, Christoph Sowada, Ewa Kocot

email: wieczorekuniversity@gmail.com

**Organizational aspects of interest**

Relation between organization of long-term care and the direction of the transition.

Relation between the organizational aspects and care transitions between the settings.

Organizational aspects that have an impact on care transitions between the settings.

- Communication among involved professional groups and its impact on care transitions between the settings
- Transfer of information and care responsibility of the patients and its impact on care transitions between the settings?
- Coordination of resources (involving nurses, pharmacists, transition care manager or program) and its impact on care transitions between the settings
- Training and education of staff have and its impact on care transitions between the settings.
- E-health and its impact on care transitions between the settings?
- Education and involvement of the patient and family and its impact on care transitions between the settings?
- Social aspects (social care)(availability of social care worker) and its impact on care transitions between the settings?

Perceptions and opinions regarding optimal care transition?

**Financial aspects of interest**

Relation between how long-term care is financed and the direction of the transition.

Relation between the financial aspects and care transitions between the settings.

- Impact of financial aspects on care transitions between the settings.
- Reimbursement of providers and impact on care transitions.
- Experiences relating to the provider reimbursement mechanism and impact on care transition.
- Impact of the rewards for the role of care coordinator on care transition.
- Relation between financial rewards and penalties and their impact on care transition.

Importance of these financial mechanisms according to the respondent.
