# Supplementary material for: Development of a tool for assessing the performance of long-term care systems in relation to care transition: Transitional Care Assessment Tool in Long-Term Care (TCAT-LTC)
Source: BMC Geriatr. 2023 Nov 20;23:760. doi: 10.1186/s12877-023-04467-z (PMC10662551; doi:10.1186/s12877-023-04467-z)
Supplement: Supplementary file 3 — Additional file 3: Appendix 3. Basic findings from the interviews. [file 12877_2023_4467_MOESM3_ESM.docx]

**Appendix 3. Basic findings from the interviews**

| **GERMANY** | |
| --- | --- |
| **Organizational aspect** | **Basic findings from the interviews** |
| Coordination of resources | - Need for better interprofessional and intersectoral collaboration among all involved in care process (PRIMARY CARE 1, HOSPITAL 2, INSURER/PAYER 1, INSURER/PAYER 2, LONG-TERM CARE 3) - Need for clear definition of responsibilities of professionals and organizations (HOSPITAL 2, HOSPITAL 1, INSURER/PAYER 2) - Need for patients’ assessments, getting to know them personally & their needs (HOSPITAL 2, HOSPITAL 1, INSURER/PAYER 2) - Importance and need for case management/manager (HOSPITAL 1, HOSPITAL 2, INSURER/PAYER 2) - Case managers role should be to focus more on patients’ and optimization of care and to look beyond the current setting (HOSPITAL 1) - Case manages could also take on social aspects (HOSPITAL 1) - Advanced practices nurses could be case managers (HOSPITAL 1) - Need for care planning and transition planning (HOSPITAL 1, INSURER/PAYER 2) - Importance of LTC infrastructure (staff etc.) (HOSPITAL 1, INSURER/PAYER 2) - Regular meetings with involved professionals and institutions (LONG-TERM CARE 2, LONG-TERM CARE 3) - Legal regulations regarding discharge planning in hospitals and disability scale (INSURER/PAYER 1) - Need for losing restrictions and reducing bureaucracy regarding hiring nurses from abroad (LONG-TERM CARE 2) - Organizations should be aware of work of other settings so that the patient is prepared prior to care transition (PRIMARY CARE 1) - System should be designed to enable professionals to adapt procedures and activities to the needs of the patients, if possible (PRIMARY CARE 1) - Adapting structures and routines for intersectoral and interprofessional collaboration (PRIMARY CARE 1) - Important positive role of the payer in planning care (HOSPITAL 2) - Visits of medical staff at the LTC facilities to avoid hospitalization (HOSPITAL 2) - Need for the support from the management (HOSPITAL 1) - Importance of implementing transitions of care models by the organization (HOSPITAL 1) - Importance of having case conferences (HOSPITAL 1) - Need for more staff with one year training, less specialized (INSURER/PAYER 1) - Discharge and transition planning should be performed together with payer (INSURER/PAYER 1) - Providing essential medicine during discharge over the weekend (LONG-TERM CARE 2) - During transition from home to LTC – patient’s family is responsible for providing information (LONG-TERM CARE 2) - Need for higher involvement of care assistants during transition process (LONG-TERM CARE 2) - Need for higher involvement of primary care (LONG-TERM CARE 2) - Availability of places to support patients & informal caregivers (LONG-TERM CARE 2) - Doctors have vital role in selecting patients’ transition destination (LONG-TERM CARE 1) - Doctors have vital role in selecting services provided to the patient in ambulatory care (LONG-TERM CARE 1) - Longer established providers are more preferred than newcomers (LONG-TERM CARE 1) |
| Limitations in coordination | - Limited availability of staff especially in LTC (HOSPITAL 2, INSURER/PAYER 1, LONG-TERM CARE 2, LONG-TERM CARE 3) - Limited availability of places in LTC facilities (HOSPITAL 2, LONG-TERM CARE 2, LONG-TERM CARE 3) - Lack of coordination between health and long-term care providers (LONG-TERM CARE 1) - Normative and legal conditions may make it difficult to delegate or transfer responsibilities (PRIMARY CARE 1) - Organizations implement their own protocols but these protocols are not adapted to the situation of the patients (PRIMARY CARE 1) - Separate financing streams for health care and long-term care (HOSPITAL 2) - Using weekends and nights by LTC facilities to transfer “difficult” patients to the hospital (HOSPITAL 2) - No care planning/transition planning means more suboptimal care transitions (HOSPITAL 1) - It is common in Germany that unclear responsibilities of the professionals lead to suboptimal transitions (HOSPITAL 1) - Case managers in Germany are focused on optimization for hospitals instead of optimization of care for the patient (HOSPITAL 1) - Limited use of case conferences (HOSPITAL 1) - Lack of legal regulations for carers from abroad (INSURER/PAYER 1) - Doctors have dominant role (INSURER/PAYER 1) - Nurses are not independent in their decisions (INSURER/PAYER 1) - Competing for the budget between medical doctors and other providers (INSURER/PAYER 1) - Limitation in division of care of staff with more training and less training (INSURER/PAYER 1) - Lack of planning regarding the location of LTC facilities (INSURER/PAYER 1) - Overproviding of ambulatory intensive LTC care (INSURER/PAYER 1) - Limited involvement of primary care (LONG-TERM CARE 2) - No formal referrals for LTC (LONG-TERM CARE 2) - Primary care physicians are overworked (LONG-TERM CARE 2) |
| Communication | - Availability of round-table regular meetings with different professionals from other settings and sectors (LONG-TERM CARE 2, LONG-TERM CARE 3, HOSPITAL 2) - Need for better communication between different professionals and sectors involved in care process (PRIMARY CARE 1, INSURER/PAYER 1, HOSPITAL 1) - Knowing personally involved professionals/institutions ease communication (HOSPITAL 2, LONG-TERM CARE 1) - Need for more communication about patients’ needs (LONG-TERM CARE 1, INSURER/PAYER 2) - Need for personal communication with patients and family (LONG-TERM CARE 1, INSURER/PAYER 2) - Importance of communication of 3 sides (sending-patient-receiving) (HOSPITAL 2) - Receiving and sending settings should communicate for planned transitions of vulnerable patients (PRIMARY CARE 1) - Importance of verbal communication (PRIMARY CARE 1) - Importance of electronic or digital ways of communication (PRIMARY CARE 1) - Need for the central actor facilitating the communication about free places in long-term care (HOSPITAL 2) - Need for on-time communication (INSURER/PAYER 2) - Communication between social care worker (sozialdienst) and LTC facilities (LONG-TERM CARE 2) - Communication between ambulatory and stationary LTC regarding patient health status (LONG-TERM CARE 2) - Good communication with the primary care (LONG-TERM CARE 3) - Need for more detailed communication (LONG-TERM CARE 3) - Limited communication between involved professionals (LONG-TERM CARE 1) |
| Limitations in communication | - Limited communication with the family (HOSPITAL 2, LONG-TERM CARE 3) - Using outdated methods (on paper) to communicate with other providers (HOSPITAL 1) - Communication with the hospitals is malfunctioning (LONG-TERM CARE 3) - Limited involvement of patient and family (LONG-TERM CARE 3) - Involved groups may not understand the information (official language/formal language) (LONG-TERM CARE 1) |
| Transfer of information and patient responsibility | - Need for electronic health information exchange, specifically electronic patient record (PRIMARY CARE 1, HOSPITAL 1, LONG-TERM CARE 2, LONG-TERM CARE 1) - Importance of standardized protocol for information exchange (HOSPITAL 1, LONG-TERM CARE 2) - Need for more detailed information (INSURER/PAYER 2, LONG-TERM CARE 3) - Importance of e-Health (PRIMARY CARE 1) - Some improvements were observed in some places in Germany, health care providers worked to develop standardized transition protocol (HOSPITAL 1) - In complex situations doctor telephone, the general practitioner for clarification (HOSPITAL 2) - Transfer of information should be going through the insurer (Plegekasse) (INSURER/PAYER 1) - Patients’ preferences should be also included (INSURER/PAYER 2) - Need for more enhanced transfer of information (LONG-TERM CARE 3) - Transfer of information is performed by social care worker (discharge manager) or by ambulatory care provider (LONG-TERM CARE 2) - Need for timely information on disability score or the patient and organization responsible for financing (LONG-TERM CARE 2) - Good transfer of information from primary care (LONG-TERM CARE 3) - Round-table meetings to exchange ideas and information (LONG-TERM CARE 3) - During transition from home to LTC – patient’s family is responsible for providing information (LONG-TERM CARE 2) |
| Limitations in transfer of information and patient responsibility | - Receiving non-specific, incomplete, delayed, or even no information is delivered (HOSPITAL 1, HOSPITAL 2, LONG-TERM CARE 3, LONG-TERM CARE 1) - Using outdated methods such as on paper or fax to transfer the information (HOSPITAL 2, HOSPITAL 1, LONG-TERM CARE 1) - Transfer of information may be affected by data protection (INSURER/PAYER 2, LONG-TERM CARE 1, HOSPITAL 1) - Need for improving transfer of information (HOSPITAL 2, LONG-TERM CARE 3) - Standardized protocol is not used in routine care (HOSPITAL 1) - Very limited use of electronic patient records (HOSPITAL 1) - Involved groups may not understand the information (official language) (LONG-TERM CARE 1) - Transfer of information is the worst with primary care providers (LONG-TERM CARE 2) |
| Education and involvement | - Patients’ and caregivers’ needs and preferences should be considered, care should be patient-centered (PRIMARY CARE 1, HOSPITAL 1, INSURER/PAYER 1, INSURER/PAYER 2) - Importance of providing information and education to the patient and caregivers (PRIMARY CARE 1, HOSPITAL 1, INSURER/PAYER 2, LONG-TERM CARE 2) - Need for involvement of patients and caregivers in decision-making process (LONG-TERM CARE 3, HOSPITAL 2, INSURER/PAYER 2) - Importance of involving caregivers in the care process (PRIMARY CARE 1, HOSPITAL 2, INSURER/PAYER 2) - Providing information and education to the patient and caregivers (HOSPITAL 2, LONG-TERM CARE 2, LONG-TERM CARE 1) - Availability of places providing advice and information (INSURER/PAYER 1, LONG-TERM CARE 2) - Regular meetings with the patients and family (HOSPITAL 2) |
| Limitations in education and involvement | - Patients’ and caregivers’ needs and preferences are not considered (PRIMARY CARE 1, HOSPITAL 1, LONG-TERM CARE 3, LONG-TERM CARE 1) - Limited involvement of patients’ and caregivers’ in decision-making (PRIMARY CARE 1, LONG-TERM CARE 3, HOSPITAL 2, HOSPITAL 1) - Informal caregivers are often poorly informed and involved (HOSPITAL 1) - Limited use of available advice, help centers by patients and caregivers (LONG-TERM CARE 2) - Decisions of patients regarding the selection of care activities may be inappropriate (LONG-TERM CARE 3) |
| Training and education of staff | - Need for well trained staff (e.g. case managers, LTC staff, care assistants) to provide high quality care (INSURER/PAYER 2, LONG-TERM CARE 2, PRIMARY CARE 1, INSURER/PAYER 1) - Staff should develop competencies to look at care from multiple perspectives, also perspective of other providers (PRIMARY CARE 1) - Professionals should have basic knowledge about how the care is organized in other settings (PRIMARY CARE 1, INSURER/PAYER 1) - Availability of well trained staff in the hospital (LONG-TERM CARE 2, HOSPITAL 2) - Mandatory training courses for the staff (LONG-TERM CARE 2, LONG-TERM CARE 3) - Change in training scheme for nurses, comprehensive training programme (improved competencies) (INSURER/PAYER 1) - More attention should be paid to the training regarding the communication/transfer of information (HOSPITAL 2) - Need for increasing awareness of the staff regarding care transition (HOSPITAL 1) - 1-year training for care assistants to provide all non-medical services (INSURER/PAYER 1) - Employers compete with each other in order to keep personnel by providing additional trainings to the staff (LONG-TERM CARE 3) |
| Limitations in training and education of staff | - Staff may not be competent enough to assess patients’ needs (PRIMARY CARE 1) - Nurses and care assistants are not trained to perform activities independently (INSURER/PAYER 1) - In the past, division within training scheme among nurses (specialized to provide care only for some specific age groups) (INSURER/PAYER 1) - Lack of psychological help/assistance provided to the staff. Need for more (LONG-TERM CARE 3) - Lack of training regarding transitional care (LONG-TERM CARE 1) |
| Telemedicine and e-Health | - Need for higher use of telemedicine and e-Health (HOSPITAL 1, PRIMARY CARE 1, INSURER/PAYER 2, LONG-TERM CARE 2, LONG-TERM CARE 3, LONG-TERM CARE 2) - Need for electronic patient record (LONG-TERM CARE 2, PRIMARY CARE 1, HOSPITAL 1, INSURER/PAYER 2) - e-Health is a mediator but not a causal factor for patient-centered care (PRIMARY CARE 1) - Importance of video consultations (HOSPITAL 1) - Importance of telemedicine, tele-nursing etc. (HOSPITAL 1) - Introducing video-consultation (INSURER/PAYER 1) - Use of health monitoring devices (LONG-TERM CARE 2) - Need for use of health monitoring devices (LONG-TERM CARE 3) |
| Limitations in telemedicine and e-Health | - Very limited use of telemedicine and e-Health (HOSPITAL 2, HOSPITAL 1, LONG-TERM CARE 1, LONG-TERM CARE 2, LONG-TERM CARE 3, LONG-TERM CARE 2, PRIMARY CARE 1) - Use among older adults can be problematic (HOSPITAL 2, HOSPITAL 1) - Very limited use of electronic patient records (HOSPITAL 1) - Limited use of telemedicine affects possibility of patients to be discharged to home (HOSPITAL 1) - Skeptical attitude of primary care towards video consultations (INSURER/PAYER 1) |
| Social care | - Social care institutions are responsible for covering the costs for LTC if patient and/or family are not capable to pay (HOSPITAL 2, LONG-TERM CARE 2, INSURER/PAYER 1) - Social care institutions help patient/caregiver to cover the costs of LTC without issues (LONG-TERM CARE 2, HOSPITAL 2) - In inpatient setting, social care workers are focused on discharge management, for example by preparing the receiving setting (PRIMARY CARE 1, LONG-TERM CARE 2) - Importance of involving social care workers in the interprofessional/intersectoral meetings (HOSPITAL 1, INSURER/PAYER 1) - The importance of good functioning discharge manager (Sozialdienst) in the hospital (INSURER/PAYER 2) - Availability of social services in nursing home and hospitals (HOSPITAL 1) - Involvement of social care worker (discharge manager) in hospital, regular meetings with providers and working on problem-solving (LONG-TERM CARE 2) - Social care institutions are interested in lowering rates for LTC facilities (INSURER/PAYER 1) |
| Limitations in social care | - Participant have mixed feelings regarding involvement of social care workers in discharge planning (PRIMARY CARE 1, HOSPITAL 1) - The role of social care workers is limited in outpatient settings (PRIMARY CARE 1) - Difficulties in communication with social care institutions (LONG-TERM CARE 3) - Unwillingness to pay by social care institutions to support LTC placement in case of cost rise (LONG-TERM CARE 3) - Social care institutions communicate with patients and/or families in incomprehensible way (LONG-TERM CARE 1) - Having separate role of social care worker in the hospital may lead to diffusion of responsibilities (HOSPITAL 1) |
| Supporting informal caregivers | - Availability of training courses for informal caregivers (HOSPITAL 2, HOSPITAL 1, LONG-TERM CARE 2, LONG-TERM CARE 3, LONG-TERM CARE 1, INSURER/PAYER 2) - Availability of respite care services (LONG-TERM CARE 2, INSURER/PAYER 1, INSURER/PAYER 2) - Patients receive cash benefits to pay to carer of their choice. Thus, informal caregiver receives financial compensation (INSURER/PAYER 1, INSURER/PAYER 2, HOSPITAL 2) - Availability of centers providing information and help to informal caregivers (LONG-TERM CARE 2, INSURER/PAYER 1) - Need for increasing the budgets for respite care (LONG-TERM CARE 2) - Importance of assessing informal caregivers’ needs (HOSPITAL 1) - Informal caregivers should also receive a training on how to take care of themselves (HOSPITAL 1) - Importance to provide information about the institutions and professionals that provide help or respite care (HOSPITAL 1) - LTC staff helps to identify patients at home that should be transitioned to formal LTC facilities (LONG-TERM CARE 1) - Payer contributes to pension insurance of informal caregivers. The level depends on disability score (INSURER/PAYER 1) |
| Limitations in supporting informal caregivers | - Sometimes offered courses and information centers are not used by informal caregivers (LONG-TERM CARE 2, LONG-TERM CARE 1) - Informal caregivers do not receive enough support during care transition, are not involved & informed sufficiently (PRIMARY CARE 1, HOSPITAL 1) - Informal caregivers do not receive ongoing support/education/information (HOSPITAL 1) - Need for more solutions regarding respite care (INSURER/PAYER 1) - Monetary compensation for respite care is prone to fraud from the applicant’s side (INSURER/PAYER 1) - Informal caregivers don’t always receive sufficient support, it depends on family’s determination (INSURER/PAYER 2) - Limited psychological support provided to informal caregivers (INSURER/PAYER 2) |
| **Financial challenges** | **Basic findings from the interviews** |
| Reimbursement | - Social care support for patients’ in reimbursement for LTC facilities in case of lack of financial funds/property/inability to pay by families (LONG-TERM CARE 2, LONG-TERM CARE 3, INSURER/PAYER 1, HOSPITAL 2) - Profitable reimbursement rates for LTC facilities & fixed and in general satisfactory salaries for staff (INSURER/PAYER 1, HOSPITAL 2) - Participants’ mixed feelings regarding value-based reimbursements (PRIMARY CARE 1, HOSPITAL 1) - Reimbursement in ambulatory LTC is satisfactory (LONG-TERM CARE 3, LONG-TERM CARE 1) - Availability of LTC insurance (HOSPITAL 2) - Importance of evidence-based reimbursements – reimbursing what works (PRIMARY CARE 1) - Two possible reimbursements for psychiatric hospital for LTC patients: DRG or per-diem (HOSPITAL 2) - Financing should be in one hand and the same across the sectors to reduce barriers between the settings (HOSPITAL 1) - Reimbursing video consultations (INSURER/PAYER 1) - Reimbursing trainings for informal caregivers (INSURER/PAYER 1) - Negotiating reimbursement rates with social care and providers (INSURER/PAYER 1) - DRG reimbursement in hospitals + budget (INSURER/PAYER 1) - Per-diem reimbursement dependent on disability score (LONG-TERM CARE 2) - The need for higher salaries for ambulatory LTC staff instead of additional rewards (LONG-TERM CARE 3) - Need for higher public funding for LTC (and reducing OOP) (LONG-TERM CARE 1) - Need for additional lump sum payment for transition period before the patients’ disability score is estimated (LONG-TERM CARE 2) |
| Limitations in reimbursement | - The role of out-of-pocket payments for LTC (INSURER/PAYER 1, LONG-TERM CARE 2, LONG-TERM CARE 3, LONG-TERM CARE 1, INSURER/PAYER 2, HOSPITAL 2) - Lack of reimbursement for interprofessional collaboration/intersectoral care/transitional care (PRIMARY CARE 1, HOSPITAL 1) - Payments per-diem may have negative impact on quality-of-care or admissions and ultimately care transitions (INSURER/PAYER 1, INSURER/PAYER 2) - Activity-based payments may have negative impact, for instance, on supplier-induced demand (HOSPITAL 1, INSURER/PAYER 1) - In Germany, reimbursement is physician-centered, focus on physician needs (PRIMARY CARE 1) - DRG reimbursement in hospital may shorten the length-of-stay without justified cause (LONG-TERM CARE 2) - Value-based payment methods – difficulty in measuring quality (HOSPITAL 1) - Payments per-diem are not flexible enough. Need for additional lump sum to compensate for variability in incurred costs (LONG-TERM CARE 2) - Extensive administrative work related to the reimbursement and reporting (HOSPITAL 2) - Reimbursement of services may be restricted to some age groups (HOSPITAL 2) - The level of reimbursement is dependent on the score on disability scale. Sometimes the disability score is provided with the delay (LONG-TERM CARE 2) - The level of reimbursement is dependent on the score on disability scale. Responsible institutions often manipulate the score for their gain (reducing costs) (LONG-TERM CARE 3) - Unwillingness to pay by social care institutions to support LTC placement (LONG-TERM CARE 3) - Reimbursement for some ambulatory LTC services don’t correspond to the needed workload (LONG-TERM CARE 3) |
| Penalties | - Penalties are not available in Germany (LONG-TERM CARE 2, LONG-TERM CARE 3, INSURER/PAYER 1) - Participant mixed feelings/hesitancy about the use of penalties (PRIMARY CARE 1, LONG-TERM CARE 2) - Too minor penalties may not have desired effect (PRIMARY CARE 1) - Penalties on their own are not sufficient measure, they need to be constructive and offer solutions (HOSPITAL 1) - Penalties could help to raise awareness about the problem (HOSPITAL 1) - No information provided by the respondent (INSURER/PAYER 2) - Penalties could be enacted for misuse, abuse, abnormalities (INSURER/PAYER 1) |
| Limitations in penalties | - Problems with appointing responsible party (LONG-TERM CARE 2, INSURER/PAYER 1) - Difficulty in measuring the quality (HOSPITAL 1) |
| Rewards | - Rewards are not available in Germany (LONG-TERM CARE 2, HOSPITAL 2, INSURER/PAYER 1) - Limited knowledge regarding rewards (HOSPITAL 1, PRIMARY CARE 1, HOSPITAL 2) - Participants’ mixed feelings/hesitancy about the use of rewards (LONG-TERM CARE 2, PRIMARY CARE 1) - Some potential for rewards to improve care transitions, stimulate practices (though participants unsure how) (HOSPITAL 1, PRIMARY CARE 1) - Additional payment during corona (LONG-TERM CARE 3) - Need for higher salaries instead of rewards (LONG-TERM CARE 3) - Importance of creating quality indicators related to care transitions (PRIMARY CARE 1) - No information provided by the respondent (INSURER/PAYER 2) |
| Limitations in rewards | - Problems with appointing responsible party (LONG-TERM CARE 2, HOSPITAL 2) - Questioning whether rewards are effective in long-term (PRIMARY CARE 1) - Difficulty in measuring the quality (HOSPITAL 1) - Problems with ‘cheating’ the system by ‘pretending’ that criteria are met (HOSPITAL 1) |

| **THE NETHERLANDS** | |
| --- | --- |
| **Organizational aspect** | **Basic findings from the interviews** |
| Communication | - Importance of good communication with the patient and informal caregiver (INSURER/PAYER, PRIMARY CARE 1, LONG-TERM CARE 1, LONG-TERM CARE 3, LONG-TERM CARE 2) - Importance of interprofessional/intersectoral communication (HOSPITAL 1, HOSPITAL 2, INSURER/PAYER, LONG-TERM CARE 1, LONG-TERM CARE 2) - Availability and importance of multidisciplinary team meetings for interprofessional/intersectoral communication (HOSPITAL 1, LONG-TERM CARE 1) - Importance of e-Health solutions to improve communication between the providers and also with the caregiver (HOSPITAL 1, INSURER/PAYER) - Important role of transfer nurses communicating with receiving setting (e.g. community care, LTC institutions) and informal caregiver (HOSPITAL 1, INSURER/PAYER) - Important role of social care workers communicating with LTC institutions, the patient and informal caregiver (HOSPITAL 1, LONG-TERM CARE 1) - Good communication between providers, especially home care and long-term care (PRIMARY CARE 1) - Professionals themselves contact the long-term care facility (PRIMARY CARE 1) - Professionals from long-term care facilities contact the person in home care to get acquainted (PRIMARY CARE 1) - Knowing professionals from the organization/institutions ease the communication (HOSPITAL 1) - For patients being discharged home without need of care, general practitioner or community physician is contacted (HOSPITAL 1) - Importance of video call or telephone call to communicate with other providers (HOSPITAL 2) - Institutions that are part of the hospital communicate easier and better (INSURER/PAYER) - Importance of good communication between the providers about patients’ medical, psychological, social and caring needs (INSURER/PAYER) - Important role of client advisors in communicating with institutions, patient and informal caregiver (LONG-TERM CARE 3) - District nurses communicate with the LTC institutions LONG-TERM CARE 3) - District nurses may also visit and communicate in person with the staff at the LTC institutions (LONG-TERM CARE 3) - Involvement of the district nurse in communication between the professionals, the patient and informal caregiver (LONG-TERM CARE 3) |
| Limitations in communication | - Sometimes interprofessional/intersectoral communication is not optimal, particularly between hospitals and home care (HOSPITAL 2, INSURER/PAYER) - Need for improvement of communication (INSURER/PAYER) |
| Transfer of information and patient responsibility | - Importance of good transfer of information between the providers/institutions and informal caregivers (HOSPITAL 1, HOSPITAL 2, LONG-TERM CARE 1, LONG-TERM CARE 2) - Importance of the quality of transferred information, for instance, completeness (PRIMARY CARE 1, HOSPITAL 2, LONG-TERM CARE 1, LONG-TERM CARE 3) - Availability of agreements between the providers may improve transfer of information (HOSPITAL 1, INSURER/PAYER) - Importance of good transfer of patients’ information including not only medical but also psychological and social aspects (LONG-TERM CARE 1, PRIMARY CARE 1) - Importance of standardized protocol for information exchange (HOSPITAL 1, LONG-TERM CARE 1) - Important role and need for electronic health records (INSURER/PAYER, LONG-TERM CARE 1) - Importance of timely transfer of information (HOSPITAL 2) - Need for transferring information to all providers involved in the next setting also including patients’ preferences (HOSPITAL 2) - If patient goes to the hospice, Information is provided with the letter and follow-up call to the GP (LONG-TERM CARE 1) - Importance of telephone call while transferring the information (LONG-TERM CARE 1) - Interprofessional collaboration may smooth the transfer of information (LONG-TERM CARE 1) - All providers should have access to agreements concerning advance care planning (LONG-TERM CARE 1) - In some cases, meeting in-person with the staff at the receiving setting may improve transfer of information (LONG-TERM CARE 3) - Good transfer of information between providers (LONG-TERM CARE 3) - Information about the patient is available in the medical file, records (LONG-TERM CARE 3) - Information about the patient is passed with the use of secured mail (LONG-TERM CARE 3) |
| Limitations in transfer of information and patient responsibility | - Sometimes transferred information is not detailed enough, is incomplete (HOSPITAL 1, HOSPITAL 2, LONG-TERM CARE 1) - Sometimes transferred information is delayed (HOSPITAL 1, HOSPITAL 2, LONG-TERM CARE 1) - Privacy laws may restrict transferring the information between the institutions (HOSPITAL 1, INSURER/PAYER) - Lack of single system for information exchange, every provider has their own system (LONG-TERM CARE 1, LONG-TERM CARE 2) - Transfer of information is one of the biggest flaws in the Netherlands (HOSPITAL 1) - Sometimes transferred information includes only one providers’ perspective (HOSPITAL 1) - Lack of participation of the provider in digital solutions to transfer information (HOSPITAL 1) - Time pressure to transfer the patient to another setting (INSURER/PAYER) - The information about the psychological aspect is often not transferred (LONG-TERM CARE 1) - Diminished responsibility of who should transfer the information (LONG-TERM CARE 1) - Provision of wrong information may affect care transition (LONG-TERM CARE 3) |
| Availability and coordination of resources | - Importance and need for better interprofessional/intersectoral collaboration among all involved in care process (PRIMARY CARE 1, HOSPITAL 1, HOSPITAL 2, LONG-TERM CARE 1, LONG-TERM CARE 2) - Availability and importance of agreements between the providers/institutions (HOSPITAL 1, LONG-TERM CARE 1, INSURER/PAYER, LONG-TERM CARE 3) - Importance of LTC infrastructure (e.g. sufficient number of staff, need for more institutions, availability of crisis beds in the nursing homes) (HOSPITAL 1, INSURER/PAYER, LONG-TERM CARE 3) - Important role of physiotherapist (HOSPITAL 1, HOSPITAL 2, INSURER/PAYER) - Importance of good patients’ assessment and indication (HOSPITAL 1, HOSPITAL 2, LONG-TERM CARE 2) - Availability and importance of multidisciplinary team meetings (HOSPITAL 1, LONG-TERM CARE 1) - Need for clear definition of responsibilities of professionals and organizations (PRIMARY CARE 1, LONG-TERM CARE 2) - Primary aim is to keep the patients as long as possible at home (LONG-TERM CARE 3, LONG-TERM CARE 2) - Availability and important role of transfer nurses (HOSPITAL 1, INSURER/PAYER) - Need for the awareness and support from the management regarding transitional care/collaboration (HOSPITAL 1, LONG-TERM CARE 2) - Importance of advanced care planning/transition planning and access to such plans by all providers (HOSPITAL 1, LONG-TERM CARE 1) - Importance of engaging community nurses during care transitions (HOSPITAL 2, LONG-TERM CARE 3) - Importance of care transition managers in the long-term care settings (INSURER/PAYER) - Need for involvement of professionals from previous setting in the next setting (e.g. long-term care facility) (PRIMARY CARE 1) - Importance of timely involvement of different professionals and timely follow-up (HOSPITAL 2) - Importance of application of transitional care interventions in regular care (HOSPITAL 2) - Need for integrating transitional care interventions in already existing care networks, not building new ones (HOSPITAL 2) - Primary care physician and nurse could have an important role within transitional care interventions (HOSPITAL 2) - Transitional care interventions should consider home as a starting point so that transitions are prevented in the first place (HOSPITAL 2) - Important role of dietician (INSURER/PAYER) - Knowing personally involved professionals/institutions (LONG-TERM CARE 1) - In urgent cases, care in nursing home is organized within few days (LONG-TERM CARE 3) - Important role of client advisors in preparing receiving setting and the client (transition from home to long-term care institutions) (LONG-TERM CARE 3) - Importance of understanding the interrelation between the reforms and impact on different actors (LONG-TERM CARE 2) - Reforms should be considering the impact on the whole system, not only on single organization (LONG-TERM CARE 2) |
| Limitations in availability and coordination of resources | - Limited availability of staff (HOSPITAL 1, LONG-TERM CARE 2) - Criteria for obtaining Wlz (indication to receive LTC care) is strict, not rational (HOSPITAL 1, LONG-TERM CARE 3) - Lack of collaboration between providers/institutions - working in silos (HOSPITAL 1, LONG-TERM CARE 2) - In some cases, waiting time, waiting list to access the next setting (LONG-TERM CARE 1, LONG-TERM CARE 3) - Waiting time for the indication Wlz that enables the patient to access long-term care home or other care institution (LONG-TERM CARE 3) - Fragmentation within the organization when it comes to responsibilities (PRIMARY CARE 1) - The size of the organization may impact the care transitions, the bigger the organization, the more difficult care transitions (PRIMARY CARE 1) - Patient moving from location A (e.g. home) to location B (e.g. nursing home) receives new physician, nurse, medication system (PRIMARY CARE 1) - New case manager introduced as a part of transitional care intervention may be unfamiliar with the patient and his/her network (HOSPITAL 2) - Certain rules and regulations may affect care transitions (INSURER/PAYER) - Reforms in one part of the system may have an unintended consequence for other involved actors (LONG-TERM CARE 2) |
| Training and education of staff | - Staff should be aware or trained about work of different professionals, in other settings (HOSPITAL 1, HOSPITAL 2, LONG-TERM CARE 3) - Importance of education regarding transitional care (INSURER/PAYER, LONG-TERM CARE 1) - Important role of multidisciplinary team meetings in getting to know about each other’s work (HOSPITAL 1, HOSPITAL 2) - Staff is well trained and educated (PRIMARY CARE 1) - Importance of educating the staff to recognize some disease specific vital signs (HOSPITAL 2) - Importance of providing additional training to staff to improve quality of care transitions (INSURER/PAYER) - Importance of training and education of staff to provide the right information to the other professionals (LONG-TERM CARE 3) - Importance of changing the mindset of professionals from “taking over” care from the patient & informal caregiver to more interaction & support-based model (LONG-TERM CARE 2) - Educating staff about the important role of providing support for self-management (LONG-TERM CARE 2) |
| Limitations in training and education of staff | - Staff having limited knowledge about work of other professionals, other settings (HOSPITAL 1) - Staff in the community setting has rather generic geriatric education and may have difficulty in dealing with complex patients with specific diseases (HOSPITAL 2) |
| Education and involvement | - Importance of well-educated and informed patient and informal caregiver (HOSPITAL 1, INSURER/PAYER, HOSPITAL 2, LONG-TERM CARE 1, LONG-TERM CARE 3, LONG-TERM CARE 2) - Importance of providing multidimensional information/education to the patient and informal caregiver (HOSPITAL 1, HOSPITAL 2, INSURER/PAYER, LONG-TERM CARE 1) - Importance of patients’ and informal caregivers’ needs and preferences (HOSPITAL 1, HOSPITAL 2, LONG-TERM CARE 1, LONG-TERM CARE 2) - Importance of the involvement of informal caregivers in the care process (HOSPITAL 2, LONG-TERM CARE 2) - Importance of assessing caregivers’ ability to provide care (HOSPITAL 2, LONG-TERM CARE 2) - Assessing informal caregivers’ ability to provide care (LONG-TERM CARE 1, LONG-TERM CARE 2) - Providing multidimensional information/education to the patient and informal caregiver (LONG-TERM CARE 3, LONG-TERM CARE 2) - Providing the patient and informal caregiver with information at an early stage (LONG-TERM CARE 3, LONG-TERM CARE 2) - Need for engagement of informal caregiver in long-term care facility (PRIMARY CARE 1) - Importance of providing the patient and informal caregiver with information and education at an early stage (HOSPITAL 1, HOSPITAL 2) - Need for addressing psychological needs of the patient and informal caregiver (INSURER/PAYER) - Professionals knowing personally involved professionals/institutions can provide more detailed information (LONG-TERM CARE 1) - Involvement of patient and informal caregiver in decision-making process (LONG-TERM CARE 1) - Providing education and support to the patient and informal caregiver for self-management (LONG-TERM CARE 2) |
| Limitations in education and involvement | - In some cases, informal caregivers are not involved in the care process, or their involvement is very limited (HOSPITAL 1, HOSPITAL 2, PRIMARY CARE 1, LONG-TERM CARE 2) - Involvement of the informal caregiver is very limited once the patient is transferred to long-term care facility (PRIMARY CARE 1) - The level of involvement of the informal caregivers depends on the organization (HOSPITAL 1) - Sometimes provided care is not patient-centered (INSURER/PAYER) - Lack of information and education to the patient may prolong recovery (INSURER/PAYER) - Some informal caregivers may be afraid/hesitant to ask questions (INSURER/PAYER) - The education and information provided to the patient and informal caregiver varies among providers/institutions (INSURER/PAYER) |
| Telemedicine and e-Health | - Important role and the use of electronic devices to monitor patients at home (HOSPITAL 2, INSURER/PAYER, LONG-TERM CARE 3, LONG-TERM CARE 2) - Important role and the need for electronic patient record that is accessible to all (HOSPITAL 1, HOSPITAL 2, INSURER/PAYER) - Important role of e-Health and telemedicine to provide optimized care transitions (LONG-TERM CARE 2, HOSPITAL 2) - Need for telemedicine and e-Health solutions to be personalized (LONG-TERM CARE 1, HOSPITAL 1) - Use of telemedicine at home, for instance medication dispenser (PRIMARY CARE 1) - The use of telemedicine is helpful in self-management (PRIMARY CARE 1) - e-Health could improve standardization (HOSPITAL 1) - Importance of providing e-Health, telemedicine resources to the patient (HOSPITAL 2) - Importance of providing e-Health and telemedicine resources to the staff (HOSPITAL 2) - Telemedicine/e-Health devices may improve communication with the family (INSURER/PAYER) - Participants mixed feelings regarding the use of telemedicine and its effectiveness (LONG-TERM CARE 1) - Need for more testing of e-health and telemedicine solutions (LONG-TERM CARE 2) |
| Limitations in telemedicine and e-Health | - The use of telemedicine stops once the patient is transferred to long-term care facility (PRIMARY CARE 1) - Lack of integration of the provider in digital solutions (HOSPITAL 1) - Privacy issues (HOSPITAL 2) - Patients’ inability to pay for electronic solutions, telemedicine (HOSPITAL 2) - The use of telemedicine among older adults is not very common (LONG-TERM CARE 1) - Older adult patients have complex needs that may not be addressed with tele-health (LONG-TERM CARE 1) - Future generations will be more digital competent due to current use of digital solutions (LONG-TERM CARE 2) |
| Social care | - Availability and important role of social care workers in hospitals and home care (PRIMARY CARE 1, HOSPITAL 1, CS, LONG-TERM CARE 1) - Important role and involvement of social care workers in preparing the transition, for instance, to nursing home (PRIMARY CARE 1, HOSPITAL 1, CS, LONG-TERM CARE 1) - Social care workers have more time to look at other aspects beyond medical care (HOSPITAL 1) - Important role of social care workers to assess the patients’ situation holistically (HOSPITAL 1) - Social care workers are not always involved in care transition (INSURER/PAYER) - Social care worker often knows the patient very well and their environment, needs and preferences (CS) - In some cases, social care worker can be seen as coordinator between different institutions (CS) - Important role of social workers in providing support to informal caregivers (LONG-TERM CARE 1) - Social care workers are well informed about different organizations providing care (LONG-TERM CARE 1) - Participant mixed feelings regarding the role of social workers (LONG-TERM CARE 3) - Social care workers can arrange volunteers (LONG-TERM CARE 3) - Social care workers may be engaged to provide support and assistance to the patient & informal caregiver, especially with non-medical tasks (LONG-TERM CARE 2) - Social care workers could socially engage the patients this could result in patients’ being more active and independent (LONG-TERM CARE 2) |
| Limitations in social care | - The role of social care worker could be performed by other professionals i.e. nurse (HOSPITAL 1, PRIMARY CARE 1) - Social components are not addressed enough (PRIMARY CARE 1) - Social care workers know patients less than a district nurse (LONG-TERM CARE 3) |
| Supporting informal caregivers | - Support provided to the informal caregivers varies among organizations/institutions (HOSPITAL 1, INSURER/PAYER) - Participant believes that provided support is sufficient (LONG-TERM CARE 3, LONG-TERM CARE 2) - Providing informal caregivers with information, guidance, and support and/or bringing them in contact with right professionals (LONG-TERM CARE 3, LONG-TERM CARE 2) - Importance and need for psychological or social support provided to the informal caregiver (HOSPITAL 2, PRIMARY CARE 1) - Need for assessing informal caregivers’ needs (PRIMARY CARE 1) - Participant doesn’t have firm opinion whether provided support to is sufficient (LONG-TERM CARE 1) - Important role of social workers in providing support to informal caregivers (LONG-TERM CARE 1) - Availability of organizations providing support to informal caregivers (LONG-TERM CARE 2) |
| Limitations in supporting informal caregivers | - Informal caregivers do not receive enough support during care transition (PRIMARY CARE 1, HOSPITAL 1) - Lack of structural involvement of informal caregivers in most settings (HOSPITAL 1) - Some informal caregivers may be afraid/hesitant to ask questions (INSURER/PAYER) |
| **Financial challenges** | **Basic findings from the interviews** |
| Reimbursement | - Need for reimbursing interprofessional/intersectoral collaboration, transitional care (PRIMARY CARE 1, HOSPITAL 2) - Importance of satisfactory salaries for the staff (INSURER/PAYER, HOSPITAL 1) - Participants mixed feelings regarding the activity-based payments (HOSPITAL 2, HOSPITAL 1) - The organization receives the budget, nurses receive salary independent of volume of care provided (HOSPITAL 1, HOSPITAL 2) - Participants limited knowledge regarding the reimbursement (LONG-TERM CARE 1, LONG-TERM CARE 3) - Importance of sufficient reimbursement level of providers/institutions (HOSPITAL 1) - Participant mixed feelings regarding value-based reimbursements (PRIMARY CARE 1) - Availability of extra quality reimbursement as a part of standard reimbursement, the effect is still unclear (INSURER/PAYER) - Importance of value-based payments and their potential to improve quality of care (HOSPITAL 1) - The government tries to keep the patients at home for as long as it is possible because it is cheaper than institutionalization (PRIMARY CARE 1) - Need for flexibility to combine reimbursement forms from the government and health insurers (PRIMARY CARE 1) - Need for increasing financing for long-term care (HOSPITAL 1) - Reimbursement per patient should be based on what is declared by the caregiver what’s needed for the patient at given moment (HOSPITAL 1) - Nurses themselves should do indication about patients’ caring needs (HOSPITAL 1) - Community nurses are financed from basic insurance (HOSPITAL 2) - Need for the reimbursement of physical therapy (HOSPITAL 2) - Five years ago Dutch government gave a lot of extra money to improve the quality of care (INSURER/PAYER) - Increasing salaries for nurses and careers increased the total number of the staff, it has an impact on time spent with patients (INSURER/PAYER) - Health insurance company dedicate additional reimbursement for training staff to improve quality of care (INSURER/PAYER) - Reimbursing long-term care organizations in advance results in possibility of organizations to secure beds, staff etc. (INSURER/PAYER) |
| Limitations in reimbursement | - The role of out-of-pocket payments for LTC (INSURER/PAYER, LONG-TERM CARE 1, LONG-TERM CARE 3, LONG-TERM CARE 2) - The reimbursement per patient is fixed according to indication, irrespective of variability in care needs, as a result some organizations may experience financial loss (HOSPITAL 1, HOSPITAL 2, LONG-TERM CARE 3) - Lack of reimbursement for interprofessional/intersectoral collaboration, for instance, when nurse from long-term care facility visit patient at home (PRIMARY CARE 1, HOSPITAL 2) - Value-based payments - Difficulty in measuring quality and keeping track of the entire transition process, need for standardized indicators (PRIMARY CARE 1, HOSPITAL 1) - Activity-based payments could have negative impact on care transition by, for instance, leading to overproduction (PRIMARY CARE 1, LONG-TERM CARE 1) - Lack of flexibility/possibility to combine reimbursement forms from the government and health insurers (PRIMARY CARE 1) - Low salaries for LTC staff, particularly community nurses (HOSPITAL 1) - Physiotherapy is not included in basic insurance (HOSPITAL 2) - Financial resources for long-term care is limited/low, this has implication on availability of beds in LTC (HOSPITAL 1) - Insufficient reimbursement for providers/institutions may affect availability/staffing levels (HOSPITAL 1) - The village or the city may be reluctant to pay for home care and may prefer moving the patients to long-term care, home care is paid by the city while long-term care is covered from tax (INSURER/PAYER) - Reimbursement for extra nurses, social care workers etc. may not be paid by insurance companies or the government even if it is meant to improve quality of care (INSURER/PAYER) - Some long-term care facilities complain about lack of financial resources to improve quality of care (INSURER/PAYER) - Not flexible reimbursement arrangements, example of earlier hospital discharge and providing care at home (INSURER/PAYER) - Cutting the budget (reform in 2015) in the Netherlands for long-term care had an impact on number of LTC settings (LONG-TERM CARE 2) - Cutting the budget for LTC organizations by the government – cuts will need to be done somewhere within the organization (LONG-TERM CARE 2) |
| Rewards | - Importance of internal motivations of staff to provide good quality care is more important than financial rewards (PRIMARY CARE 1, INSURER/PAYER, LONG-TERM CARE 1, LONG-TERM CARE 3) - Participant mixed feelings regarding the use of financial rewards (PRIMARY CARE 1, INSURER/PAYER, LONG-TERM CARE 2) - Financial rewards at the organizational level could improve quality of care by, for instance, encouraging collaboration between professionals/providers/sectors (HOSPITAL 1, INSURER/PAYER) - Participants negative feelings towards the use of financial rewards (LONG-TERM CARE 3) - Rewarding organizations once a year based on their performance (HOSPITAL 1) - Financial rewards could be potentially reinvested by the organizations to further improve quality of care (i.e. staff, beds, education) (HOSPITAL 1) - Rewards should be for organizations, not for individuals (HOSPITAL 1) - System of rewards was used in the Netherlands (INSURER/PAYER) - Stimulation is more effective than rewards. Money should be put into developing competencies of staff so that the effect is maintained (INSURER/PAYER) - Financial rewards have potential to impact care transition, but on organizational level, not at healthcare professional level (LONG-TERM CARE 1) - Lack of financial rewards for district nurses (LONG-TERM CARE 3) |
| Limitations in rewards | - Financial rewards are short term stimulation (LONG-TERM CARE 2, INSURER/PAYER) - Having rewards for long-term is not possible as someone would need to pay for it (INSURER/PAYER) - Once reward system stops, the efforts to improve quality of care also stop (the ‘extra’ also stops) (INSURER/PAYER) |
| Penalties | - Participant mixed feelings regarding the use of penalties (PRIMARY CARE 1, HOSPITAL 1, HOSPITAL 2, LONG-TERM CARE 2) - Participants negative feelings regarding the use of penalties (INSURER/PAYER) - Availability of benchmarking, getting less or more money based on performance (PRIMARY CARE 1) - Need for better indicators for penalties, based more on outcomes that are important for the patient, and nurses (PRIMARY CARE 1) - Penalties could be issued for inappropriate care, referral, bad communication, transfer of information or delayed care etc.(HOSPITAL 2) - System of penalties is introduced in the Netherlands (INSURER/PAYER) - Financial penalties have potential to impact care transition, but on policy level (LONG-TERM CARE 1) - Lack of financial penalties for community nurses (LONG-TERM CARE 3) - Internal motivations of staff to provide good quality care is more important than financial penalties (LONG-TERM CARE 3) |
| Limitations in penalties | - Problems with appointing responsible party (HOSPITAL 1, HOSPITAL 2) - Financial penalties could have an impact on admission policy, for instance, by admitting healthier patients (LONG-TERM CARE 1) - Problems with complexity of the patients (HOSPITAL 2) - The effect of penalties is short-lived (INSURER/PAYER) - Penalties could be harmful and negatively affect quality of care (INSURER/PAYER) - Penalties don’t work (INSURER/PAYER) - Problems with measuring the quality of care (LONG-TERM CARE 2) - There are always individuals who try to “cheat” the system (LONG-TERM CARE 2) |

| **POLAND** | |
| --- | --- |
| **Organizational challenges** | **Basic findings from the interviews** |
| Coordination of resources | - Need for coordinator (PRIMARY CARE 2, HOSPITAL 2, INSURER/PAYER, LONG-TERM CARE 2, LONG-TERM CARE 1) - Need for development of LTC infrastructure & resources (beds, facilities, staff) (HOSPITAL 1, INSURER/PAYER, LONG-TERM CARE 2, LONG-TERM CARE 1, PRIMARY CARE 1) - Important role of physiotherapists/rehabilitation (HOSPITAL 2, HOSPITAL 1, LONG-TERM CARE 2, LONG-TERM CARE 1) - Coordinator should be linked to community, know environment, the patient etc. (PRIMARY CARE 2, HOSPITAL 2, INSURER/PAYER, PRIMARY CARE 1) - Important role of primary care and need for stronger involvement (PRIMARY CARE 2, HOSPITAL 1, PRIMARY CARE 1) - Need for developing binding procedures/regulations regarding the transition/care coordination (LONG-TERM CARE 2, LONG-TERM CARE 1, PRIMARY CARE 1) - Important role of care assistants (PRIMARY CARE 1, HOSPITAL 1) - Important role of nurses (PRIMARY CARE 2, PRIMARY CARE 1) - Important role of charities and volunteers (INSURER/PAYER, PRIMARY CARE 1) - Need for timely provision of LTC (LONG-TERM CARE 1, PRIMARY CARE 1) - Need for shortening the waiting time for LTC (LONG-TERM CARE 1, PRIMARY CARE 1) - Need for better classification of patients according to needs (considering the patient & environment) (HOSPITAL 1, PRIMARY CARE 1) - Need for coordination of financial resources between the health and social system (INSURER/PAYER) - Medical staff, specifically general practitioner should not be involved in care coordination due to shortage (INSURER/PAYER) - Coordinator could be public health graduate, paramedic or a nurse (INSURER/PAYER) - It is important to consider the resource management efficiency in order to deliver effective care to higher number of patients (INSURER/PAYER) - Questioning the relevance of care coordinator only in the inpatient settings (INSURER/PAYER) - Coordination of resources is the most important (INSURER/PAYER) - Need for 24/7 availability of doctors in LTC facilities (LONG-TERM CARE 2) - Availability of better medications in hospitals than in LTC (LONG-TERM CARE 2) - Need for more (multidisciplinary) staff in LTC facilities (LONG-TERM CARE 2) - Regulations regarding the kind of staff in LTC facilities (LONG-TERM CARE 2) - Need for addressing multiple aspects at once – medical, psychological, social, spiritual (LONG-TERM CARE 2) - Care transition should be coordinated from the beginning till the end (LONG-TERM CARE 2) |
| Limitations in coordination | - Limited availability of places in LTC facilities (INSURER/PAYER, LONG-TERM CARE 1, HOSPITAL 1) - Limited availability of LTC staff to provide care at home & LTC facilities (LONG-TERM CARE 2, HOSPITAL 1, LONG-TERM CARE 1) - Lack of binding procedures/regulations regarding the transition (LONG-TERM CARE 2, LONG-TERM CARE 1, PRIMARY CARE 1) - Lack of coordination between providers (HOSPITAL 2, HOSPITAL 1, LONG-TERM CARE 1) - Long waiting time to access LTC facilities (PRIMARY CARE 2, LONG-TERM CARE 1) - Limited involvement of primary care (HOSPITAL 1, LONG-TERM CARE 2) - Lack of coordination between the health and the social system (INSURER/PAYER, LONG-TERM CARE 2) - Long waiting times for specialized care (PRIMARY CARE 2) - Lack of transitional care coordinator (PRIMARY CARE 2) - Volunteers may not want to perform caring tasks (HOSPITAL 1) - Volunteers’ rotation (HOSPITAL 1) - Limited staff in hospitals (LONG-TERM CARE 2) - In hospitals focus on medical care only (LONG-TERM CARE 2) - Not enough settings helping in care transition (LONG-TERM CARE 1) - Insufficient number of social care workers (LONG-TERM CARE 1) - Infrastructure of some LTC facilities is not functional, adapted (LONG-TERM CARE 1) |
| Communication | - Need for better communication between professionals representing different providers and sectors (LONG-TERM CARE 2, LONG-TERM CARE 1) - Limited or lack of communication with the patient/family regarding discharge (LONG-TERM CARE 2, PRIMARY CARE 1) - Importance of good communication between the providers about patients’ needs (PRIMARY CARE 1) - Need for communication with the family (PRIMARY CARE 1) - Need for active communication between engaged professional groups (LONG-TERM CARE 2) - Need for timely communication between sending-receiving setting (LONG-TERM CARE 1) - Detailed information provided by hospitals to LTC (HOSPITAL 1) - Telephone calls to patients should be introduced (INSURER/PAYER) - Short telephone communication between hospital and LTC facility (INSURER/PAYER) - Lack of communication with the primary care physicians (INSURER/PAYER) - Important role of social care workers in communication between LTC facilities (LONG-TERM CARE 2) |
| Limitations in communication | - Limited/very limited communication between providers (PRIMARY CARE 2, HOSPITAL 2, HOSPITAL 1, LONG-TERM CARE 2, INSURER/PAYER, LONG-TERM CARE 1, PRIMARY CARE 1) - No direct communication between providers (PRIMARY CARE 2, HOSPITAL 2) - Limited communication between the staff and patient/family during discharge (LONG-TERM CARE 2, PRIMARY CARE 1) - Short telephone communication between hospital and LTC facility (INSURER/PAYER) - Lack of communication with the primary care (INSURER/PAYER, PRIMARY CARE 1) |
| Transfer of information and patient responsibility | - Need for structured/standardized information exchange, especially between the hospital and primary care (PRIMARY CARE 2, HOSPITAL 1, LONG-TERM CARE 2) - Transfer of documents done by hospital is more accurate than in primary care (HOSPITAL 1) - Nurses should be engaged (HOSPITAL 2) - Providers receive documents earlier to prepare LTC setting for the patient (HOSPITAL 1) - Complete information provided by the hospitals to LTC (HOSPITAL 1) - In hospital social care workers are responsible for transfer of information to LTC facilities (HOSPITAL 1) - Need for introduction of online platform to transfer the patient’s information – digitalization (INSURER/PAYER) - Transferring full medical information from LTC facility to the hospital (LONG-TERM CARE 2) - Information card follows the patient (LONG-TERM CARE 2) - Need for timely transfer of information between sending-receiving setting, arranging place (LONG-TERM CARE 1) |
| Limitations in transfer of information and patient responsibility | - Patient carrying the information (PRIMARY CARE 2, HOSPITAL 1, INSURER/PAYER, LONG-TERM CARE 2) - Very limited, not-detailed transfer of information (HOSPITAL 2, LONG-TERM CARE 2, PRIMARY CARE 1) - Limited use of the online platform to transfer the information (PRIMARY CARE 2) - Lack of structured information exchange between hospital and primary care (PRIMARY CARE 2) - Very limited transfer of information (HOSPITAL 2) - Lack of direct contact with the other provider (HOSPITAL 2) - Outdated transferring of information on paper (INSURER/PAYER) - Making regulations regarding the need to prepare discharge letters may further burden limited staff (INSURER/PAYER) - Lack of information (to the LTC) regarding the resident/patient admitted to the hospital (LONG-TERM CARE 2) - Problem with the transfer of the information card within the hospital (single setting) (LONG-TERM CARE 2) - Discharge letters are not standardized (LONG-TERM CARE 2) |
| Education and involvement | - Preparing and providing education to the patient and/or caregiver, not only in the hospital but also at home (PRIMARY CARE 2, HOSPITAL 2, HOSPITAL 1, PRIMARY CARE 1) - Need for provision of multidimensional information/education to the patient and the family (HOSPITAL 2, INSURER/PAYER, LONG-TERM CARE 2, PRIMARY CARE 1) - Providing medical and organizational advice by nurses in primary care settings (PRIMARY CARE 2) - Family’s knowledge and involvement play crucial role (HOSPITAL 2, HOSPITAL 1) - Availability of the program to educate the family (HOSPITAL 2) - Patient and family readiness for the transition (HOSPITAL 2) - Patient and family has the right to receive the information in the hospital (HOSPITAL 1) - District nurse provide education to the family (HOSPITAL 1) - Family is responsible for the patient (HOSPITAL 1) - Need for the program directed to informal caregivers and their needs (LONG-TERM CARE 2) - Availability of courses educating informal caregivers (LONG-TERM CARE 1) - Availability of information in forms of leaflets and instructional videos (PRIMARY CARE 1) |
| Limitations in education and involvement | - Patient/caregiver lack of preparedness (HOSPITAL 2, INSURER/PAYER) - Patient/caregiver limited knowledge and need for informational support (INSURER/PAYER, LONG-TERM CARE 2) - Staff not obliged to provide support (INSURER/PAYER, HOSPITAL 2) - Short, unstructured information provided to the patient & caregivers (PRIMARY CARE 2) - Older patients’ impairment (HOSPITAL 1) - Family’s unwillingness to be involved in care (HOSPITAL 1) - Lack of coordinator that would inform the patient and the caregiver(INSURER/PAYER) - Nurses are not trained to inform the patients about the care in other settings (INSURER/PAYER) |
| Training and education of staff | - Need for trainings/education of staff (PRIMARY CARE 2, HOSPITAL 2, LONG-TERM CARE 2, LONG-TERM CARE 1) - Availability of trainings regarding geriatric/LTC (HOSPITAL 2) - Availability of trainings for care workers – care assistants for patients with caring needs (HOSPITAL 1) - Need for providing additional trainings to the care coordinators (if available) (INSURER/PAYER) - Physicians are trained with regard to patient’s information card (INSURER/PAYER) |
| Limitations in training and education of staff | - Knowledge of staff regarding the organization of LTC system is limited (LONG-TERM CARE 2, INSURER/PAYER) - Young medical staff do not poses knowledge about transitional care (HOSPITAL 2) - No training for nurses on care provision in other settings (INSURER/PAYER) |
| Telemedicine and e-Health | - Possibility to monitor some patient groups (HOSPITAL 1, INSURER/PAYER, LONG-TERM CARE 2, LONG-TERM CARE 1, PRIMARY CARE 1) - Availability of telephone consultations, tele-information (PRIMARY CARE 2, LONG-TERM CARE 2, LONG-TERM CARE 1, PRIMARY CARE 1) - Usefulness of video consultations (PRIMARY CARE 2, INSURER/PAYER) - Tele-information enables to access the information instantly and remotely (LONG-TERM CARE 2, LONG-TERM CARE 1) - Introduction of online platform to transfer the patient’s information (PRIMARY CARE 2) - Need for introduction of online platform to transfer the patient’s information - digitalization (INSURER/PAYER) - Usefulness of telephone consultations to provide referrals (PRIMARY CARE 2) - E-health may improve communication among parties (PRIMARY CARE 2) |
| Limitations in telemedicine and e-Health | - Limited use of telemedicine and e-health (HOSPITAL 1, PRIMARY CARE 1) - Older adults prefer to avoid using telemedicine (HOSPITAL 2) - Limited use of telemedicine and e-health (HOSPITAL 1) - Long-term use of telemedicine is not beneficial (HOSPITAL 1) - Telemedicine is for physically fit patients (HOSPITAL 1) - The use of technologies may use scare resources without actual proof it will work (INSURER/PAYER) - Lack of funding for telemedicine, rather episodic (LONG-TERM CARE 1) |
| Social care | - Need for proactive engagement and involvement of social care workers (PRIMARY CARE 2, HOSPITAL 2, HOSPITAL 1, LONG-TERM CARE 2, PRIMARY CARE 1) - Social care workers are responsible for preparing documents and communicating with LTC facilities (HOSPITAL 2, HOSPITAL 1, LONG-TERM CARE 2, PRIMARY CARE 1) - Need for social care workers to provide holistic care and support (HOSPITAL 1, INSURER/PAYER, LONG-TERM CARE 2) - Availability of social workers seem to be high (HOSPITAL 2) - Need for creating the system of delivering hot meals, socialization and monitoring (INSURER/PAYER) - Need for social care workers that prepare the receiving setting and family (LONG-TERM CARE 2) |
| Limitations in social care | - Insufficient number of social care workers (HOSPITAL 1, LONG-TERM CARE 1, INSURER/PAYER) - Some social care workers are unresponsive, disorganized (LONG-TERM CARE 2) - Patients and caregivers do not know how to access help (HOSPITAL 2) - Social care worker tasks are limited to administrative role (HOSPITAL 1) - Patient’s income may limit access to social care (HOSPITAL 1) - Lack of social coordinator (INSURER/PAYER) - Needs of older adults to socialize and thus unnecessary doctors’ visits (INSURER/PAYER) - Lack of the person checking suitability of the home for the discharged patient (INSURER/PAYER) - Competencies of social care workers are very limited (LONG-TERM CARE 1) - Lack of coordination between health and social care (INSURER/PAYER) - Limited number of hospitals with social care workers, if social care worker is unavailable, patients need to organize the care by themselves (PRIMARY CARE 1) |
| Supporting informal caregivers | - Need for educational/informational/instrumental support (LONG-TERM CARE 2, HOSPITAL 2) - Need for the coordinator that would guide the patient and the family throughout the entire process. Provide medical, administrative, legal support (LONG-TERM CARE 2, INSURER/PAYER) - Need for monitoring patients’/family needs (HOSPITAL 2) - Some minor help/advice provided by the doctors (INSURER/PAYER) - Social care workers that prepare the receiving setting and family (LONG-TERM CARE 2) - Need for respite care services (LONG-TERM CARE 2) - Availability of trainings for informal caregivers (LONG-TERM CARE 1) |
| Limitations in supporting informal caregivers | - Very limited or even no support to the informal caregivers (HOSPITAL 1, INSURER/PAYER, LONG-TERM CARE 2, HOSPITAL 2) - Patients and family need to search for support/information/help by their own (HOSPITAL 1, LONG-TERM CARE 2, LONG-TERM CARE 1, HOSPITAL 2) - Supporting caregivers and the patient is not mandatory (LONG-TERM CARE 2, INSURER/PAYER) - Short, unstructured information for the patient/family (PRIMARY CARE 2) - Lack of system providing respite care (INSURER/PAYER) - Providing support is episodic, depending on the funding (LONG-TERM CARE 1) |
| **Financial challenges** | **Basic findings from the interviews** |
| Reimbursement | - Need for higher reimbursement of LTC facilities (HOSPITAL 1, LONG-TERM CARE 2, LONG-TERM CARE 1, INSURER/PAYER) - Need for competitive/higher salaries for LTC staff (HOSPITAL 1, LONG-TERM CARE 2, LONG-TERM CARE 1, PRIMARY CARE 1) - Need for reimbursement for coordination/coordinator (if available) that is satisfactory (HOSPITAL 2, INSURER/PAYER, PRIMARY CARE 2) - Important role of charities, NGOs, EU and volunteers in providing financial support (PRIMARY CARE 1, LONG-TERM CARE 2, LONG-TERM CARE 1 ) - Need for higher number of contracts for nurses providing LTC (PRIMARY CARE 1) - Introducing additional reimbursement for uploading patients’ information on the online platform (PRIMARY CARE 2) - Fundholding in primary care may improve care e.g., shorten waiting list to the specialist (PRIMARY CARE 2) - Help from the government to cover the costs of LTC (HOSPITAL 2) - Respondents’ very limited knowledge regarding financing (HOSPITAL 2) - Reimbursement for individual patient care should be according to the resources used, not according to disability (HOSPITAL 1) - Need for coordination of financial resources between the health and social system (INSURER/PAYER) - Perhaps budgets should be used to pay for transitional care? (INSURER/PAYER) - Perhaps introducing degressive payment system, financing per person-day in LTC (INSURER/PAYER) - Need for financing programs supporting informal caregivers (LONG-TERM CARE 2) - Potential solution - development of LTC wards next to hospital - lower reimbursement than hospital (LONG-TERM CARE 1) - Financing day care home as a local government (LONG-TERM CARE 1) - Need for reimbursement of care homes by NFZ (LONG-TERM CARE 1) - Need for additional reimbursement beside per capita payment for family nurses for additional services provided (PRIMARY CARE 1) - Need for additional quality-based payments (PRIMARY CARE 1) |
| Limitations in reimbursement | - The role of out-of-pocket payments for LTC (HOSPITAL 2, HOSPITAL 1, LONG-TERM CARE 2, LONG-TERM CARE 1, PRIMARY CARE 1) - Low salaries for LTC staff (HOSPITAL 1, LONG-TERM CARE 2, LONG-TERM CARE 1, PRIMARY CARE 1) - Low reimbursements/underestimated contracts for LTC facilities (HOSPITAL 1, INSURER/PAYER, LONG-TERM CARE 2) - Introducing activity-based payments may affect quality (INSURER/PAYER, LONG-TERM CARE 1, PRIMARY CARE 1) - Fee-for-service may lead to overtreatment (INSURER/PAYER) - Activity based payments for nurses could affect quality of services provided (PRIMARY CARE 1) - Fixed contracts with LTC facilities (HOSPITAL 1, LONG-TERM CARE 2) - No separate reimbursement for transitional care (PRIMARY CARE 2, INSURER/PAYER) - Very low reimbursement for the care coordinator (PRIMARY CARE 2) - Not enough contracts for health staff to provide care at home (LONG-TERM CARE 2) - Quality-based reimbursements - Difficulty in measuring quality (INSURER/PAYER) - Capitation may lead to overuse of services, unnecessary care transitions to specialized care (PRIMARY CARE 2) - Unwillingness to contribute by commune to LTC costs (HOSPITAL 1) - Patient’s income - social care may refuse to help (HOSPITAL 1) - LTC facilities receive more money for sicker patients = they are preferred over healthier ones (HOSPITAL 1) - Separate reimbursement mechanism for the health and the social system (INSURER/PAYER) - Flat-rate payments may lead to longer hospitalizations (INSURER/PAYER) - Lack of government funding for telemedicine, rather episodic (LONG-TERM CARE 1) - Out-of-pocket payments for telemedicine (LONG-TERM CARE 1) - Lack of government funding for courses for caregivers, rather episodic (LONG-TERM CARE 1) - Additional funding’s streams may be episodic (LONG-TERM CARE 1) - Lack of reimbursement of day care homes from National Health Fund (LONG-TERM CARE 1) - Need for satisfactory salary for the care coordinator if such role is introduced (PRIMARY CARE 1) |
| Penalties | - Penalties could be issued for inappropriate care, adverse events, different kind of abuse, misuse, abnormalities or for not fulfilling the contract, unnecessary referrals and lack of vital information in the referrals (PRIMARY CARE 2, LONG-TERM CARE 2, PRIMARY CARE 1, HOSPITAL 1, LONG-TERM CARE 1) - Existence of penalties is a necessity (LONG-TERM CARE 1) - Respondent unfamiliar with penalties (HOSPITAL 2) - Penalties could be issued for care coordinator, if the role of care coordinator exists (HOSPITAL 2) - Lack of penalties regarding transitional care (INSURER/PAYER) - Penalties should be constructive (LONG-TERM CARE 2) - Penalties should be symbolic (LONG-TERM CARE 2) |
| Limitations in penalties | - Penalties could be harmful (LONG-TERM CARE 2, LONG-TERM CARE 1, HOSPITAL 2) - Difficulty in measuring quality of care (HOSPITAL 2, INSURER/PAYER) - Penalties are sometimes inadequate (LONG-TERM CARE 2) - Difficulty in estimating responsibility if something goes wrong (PRIMARY CARE 1) - The use of penalties is questionable (PRIMARY CARE 1) |
| Rewards | - Need for introduction of rewards (LONG-TERM CARE 1, HOSPITAL 2, LONG-TERM CARE 2, PRIMARY CARE 2) - Coordinator (if available) should be eligible for rewards (HOSPITAL 2, LONG-TERM CARE 2) - Additional activities that improve quality of care should be rewarded (LONG-TERM CARE 2) - May improve the care (PRIMARY CARE 2) - Rewards must be satisfactory (PRIMARY CARE 2) - Respondent unfamiliar with rewards (HOSPITAL 2) - Rewards do not exist in the hospital (HOSPITAL 1) |
| Limitations in rewards | - No rewards available (LONG-TERM CARE 2, LONG-TERM CARE 1, HOSPITAL 1) - Problem with measuring quality of care (INSURER/PAYER) - The use of rewards is questionable (PRIMARY CARE 1) |
