# Supplementary material for: Development of a tool for assessing the performance of long-term care systems in relation to care transition: Transitional Care Assessment Tool in Long-Term Care (TCAT-LTC)
Source: BMC Geriatr. 2023 Nov 20;23:760. doi: 10.1186/s12877-023-04467-z (PMC10662551; doi:10.1186/s12877-023-04467-z)
Supplement: Supplementary file 4 — Additional file 4: Appendix 4. Transtitional Care Assessment Tool (TCAT) – Relevance of the items. [file 12877_2023_4467_MOESM4_ESM.docx]

**Appendix 4**

**Transtitional Care Assessment Tool (TCAT) – Relevance of the items**

| **Category/sub-category** | **Indicator** | **Explanation** | **Relevance** | | | |
| --- | --- | --- | --- | --- | --- | --- |
|  |  |  | **Very relevant** | **Somehow relevant** | **Not relevant** | **I don’t have opinion/I don’t know** |
| **Organizational aspects** | | | | | | |
| **1. Communication** | 1.1 The use of interprofessional meetings within one setting in specific complex cases | Are different professionals from one setting meeting (e.g., in the form of round-table meetings) to discuss patients’ case in specific complex cases? | 1 expert (20%) | 4 experts (80%) |  |  |
|  | 1.2 Direct communication between different providers | Are providers in personal contact regarding planned/during care transition? (e.g., hospital – primary care, long-term care – hospital, long-term care - social care, ambulatory long-term care – stationary long-term care). Consider all forms of communication (e.g., verbal, written, digital) | 3 experts (60%) | 1 expert (20%) | 1 expert (20%) |  |
|  | 1.3 On time communication | Is communication between providers on time and without delay? | 4 experts (80%) |  |  | 1 expert (20%) |
|  | 1.4 Communication of providers and institutions (if needed) | Is there communication between providers and institutions (including payers/insurers/organizers of long-term care) regarding patient’s case? | 3 experts (60%) | 2 experts (40%) |  |  |
|  | 1.5 Communication of 3 sides (sending-patient/ informal caregiver-receiving) | Is patient and/or informal caregiver involved in communication between sending and receiving setting? | 4 experts (80%) |  | 1 expert (20%) |  |
| **2.Transfer of information** | 2.1 Standardized/structured discharge information | Is discharge information standardized/structured? | 5 experts (100%) |  |  |  |
|  | 2.2 Completness of transferred information | Is transferred information complete and includes all essential information? | 5 experts (100%) |  |  |  |
|  | 2.3 Timeliness of transferred information | Is transfer of information on time? | 5 experts (100%) |  |  |  |
|  | 2.4 Responsibility for transferring information | Is there an individual responsible for transferring information? Is there a person who can be contacted in case of any issue? | 4 experts (80%) | 1 expert (20%) |  |  |
|  | 2.5 Transferring information regarding patients’ and/or informal caregivers’ preferences | Are patients’ and/or informal caregiver preferences included in the transferred information?  (e.g., preferences concerning long-term care placement, medical treatment, activities of daily life living) | 3 experts (60%) |  |  | 2 experts (40%) |
| **3. Availability & Coordination of resources** | 3.1 Number of beds in LTC facilities | Is number of beds in different LTC facilities sufficient to address older population needs? | 3 experts (60%) |  | 2 experts (40%) |  |
|  | 3.2 Number of staff in LTC | Is number of staff sufficient in LTC to address older population needs? | 2 experts (40%) | 1 expert (20%) | 2 experts (40%) |  |
|  | 3.3 Waiting time for LTC | Are patients able to access LTC without waiting time? | 4 experts (80%) |  | 1 expert (20%) |  |
|  | 3.4 Number of social care workers | Is number of social care workers sufficient to address older population needs? | 2 experts (40%) | 2 experts (40%) | 1 expert (20%) |  |
|  | 3.5 Regular meetings of involved providers/institutions | Are there regular meetings of stakeholders - including all providers from health care and social system, organizers, payers, involved in the transition process? | 1 expert (20%) | 2 experts (40%) | 2 experts (40%) |  |
|  | 3.6 Availability and involvement of care coordinator | Is there care coordinator available? Is care coordinator actively involved especially in complex cases? | 3 experts (60%) | 1 expert (20%) | 1 expert (20%) |  |
|  | 3.7 Coordinated discharge process by sending - receiving party | Is discharge process coordinated between sending – receiving party? | 4 experts (80%) |  |  | 1 expert (20%) |
|  | 3.8 Access to physiotherapists/rehabilitation | Is there an access to physiotherapists/rehabilitation in a setting, for instance, primary care, hospital, long-term care? | 1 expert (20%) | 3 experts (60%) | 1 expert (20%) |  |
|  | 3.9 Involvement of primary care | Is primary care involved in patients’ care at all levels/in all settings? | 1 expert (20%) | 4 experts (80%) |  |  |
|  | 3.10 Assessing informal caregivers’ ability to provide appropriate care (if applicable) | Is informal caregivers’ ability to provide appropriate care required at home assessed? | 4 experts (80%) | 1 expert (20%) |  |  |
| **4. Training and education of staff** | 4.1 Availability of trainings regarding transitional care | Are there trainings regarding general aspects of transitional care available for staff? | 3 experts (60%) | 1 expert (20%) |  | 1 expert (20%) |
|  | 4.2 Availability of trainings provided to case managers/care coordinators (if applicable) | Are there specialized trainings for case managers/care coordinators available? | 2 experts (40%) | 2 experts (40%) |  | 1 expert (20%) |
|  | 4.3 Availability of trainings for care assistants (if applicable) | Are there trainings on how to provide care to older patient available for care assistants? | 2 experts (40%) | 2 experts (40%) |  | 1 expert (20%) |
|  | 4.4 Obligation to uptake additional courses/trainings | Is there an obligation for staff to uptake additional courses/trainings regarding transitional care? | 2 experts (40%) | 1 expert (20%) | 1 expert (20%) | 1 expert (20%) |
| **5. Education/support of the patient/informal caregivers** | 5.1 Access to education/advise/information for patient and/or informal caregivers (related mostly to medical & caring needs) | Is there availability of places to educate/advise/inform patient and/or informal caregivers? Consider education/advise/information relating mostly to medical & caring needs. | 3 experts (60%) | 1 expert (20%) | 1 expert (20%) |  |
|  | 5.2 Access to information (related to administrative/organizational aspects) | Is there availability of essential information (including administrative/organizational information) provided to the patient and/or informal caregivers? | 4 experts (80%) | 1 expert (20%) |  |  |
|  | 5.3 Access to instrumental support | Do patient/informal caregiver have access to instrumental support (e.g. in form of material goods – wheelchairs, adjustable beds, services or task assistance) or at least support in finding these resources? | 3 experts (60%) | 2 experts (40%) |  |  |
|  | 5.4 Access to coordinator guiding through the transition process | Do patient and/or informal caregiver have access to coordinator that guide them through the transition process? | 3 experts (60%) | 1 expert (20%) |  | 1 expert (20%) |
| **6. Involvement of the patient/informal caregiver** | 6.1 Involving patient & informal caregiver in decision-making process | Is patient & informal caregiver involved in decision-making process? | 4 experts (80%) | 1 expert (20%) |  |  |
|  | 6.2 Considering patients’ preferences, if possible | Are patients’ preferences considering transition considered? | 4 experts (80%) |  | 1 expert (20%) |  |
|  | 6.3 Considering informal caregivers’ preferences, if possible | Are informal caregivers’ preferences considering transition considered? | 4 experts (80%) |  | 1 expert (20%) |  |
| **7. Supporting informal caregivers** | 7.1 Reimbursement of trainings/courses for informal caregivers | Are free trainings/courses for informal caregivers funded? | 2 experts (40%) | 1 expert (20%) | 1 expert (20%) | 1 expert (20%) |
|  | 7.2 Access to respite care services | Is there an access to respite care services for informal caregivers? | 4 experts (80%) | 1 expert (20%) |  |  |
|  | 7.3 Financial renumeration of informal caregivers | Are informal caregivers financially compensated for providing care? | 2 experts (40%) | 1 expert (20%) | 1 expert (20%) | 1 expert (20%) |
| **8. Telemedicine and e-Health** | 8.1 Access to electronic patient record | Is there an access to electronic patient record in all settings? | 4 experts (80%) | 1 expert (20%) |  |  |
|  | 8.2 The use of medical technologies, e-Health to monitor patients’ health | Are medical technologies used to monitor patients’ health? | 1 expert (20%) | 3 experts (60%) | 1 expert (20%) |  |
|  | 8.3 Availability of telephone consultations | Are telephone consultations available to patients and/or informal caregivers? (refers to the medical conslutation) | 2 experts (40%) | 2 experts (40%) | 1 expert (20%) |  |
|  | 8.4 Availability of video consultations | Are video consultations available to patients and/or informal caregivers? | 1 expert (20%) | 3 experts (60%) | 1 expert (20%) |  |
|  | 8.5 Access to tele-information | Do patients/ informal caregivers have access to tele-information? | 2 experts (40%) | 1 expert (20%) | 1 expert (20%) | 1 expert (20%) |
| **9. Social care** | 9.1 Involvement of social care workers to look after the patient | Are social care workers actively involved to look after the patient at home? | 1 expert (20%) | 3 experts (60%) |  | 1 expert (20%) |
|  | 9.2 Social care worker involvement in discharge process (in hospital) | Are social care workers involved in discharge process? (In collaboration with health care worker) | 2 experts (40%) | 2 experts (40%) |  | 1 expert (20%) |
|  | 9.3 Social care worker prepares patient & informal caregiver | Does social care worker prepare patient & informal caregiver before the discharge? (In collaboration with health care worker) | 2 experts (40%) | 2 experts (40%) |  | 1 expert (20%) |
|  | 9.4 Social care worker prepares receiving setting | Does social care worker prepare receiving setting before the discharge? (In collaboration with health care worker) | 2 experts (40%) | 1 expert (20%) | 1 expert (20%) | 1 expert (20%) |
|  | 9.5 Social care worker competencies and responsibilities | Do social care worker competencies and responsibilities allow for proactive engagement in care coordination? | 3 experts (60%) | 1 expert (20%) | 1 expert (20%) |  |
| **Financial aspects** | | | | | | |
| **10. Primary care** | 10.1 Appropriateness of reimbursement level - sufficient reimbursement level to cover the costs? | Is the reimbursement level sufficient to cover the costs incurred? | 2 experts (40%) | 3 experts (60%) |  |  |
|  | 10.2 Presence of incentives that stimulate cost-efficient care | Are there any incentives (e.g. Pay for Performance, Pay for Quality etc.) in place to stimulate cost-efficient care? | 2 experts (40%) | 2 experts (40%) |  | 1 expert (20%) |
|  | 10.3 Sufficient renumeration level of the staff | Is the renumeration for staff sufficient to ensure the retention of staff? | 2 experts (40%) | 2 experts (40%) |  | 1 expert (20%) |
|  | 10.4 Compensation for care coordinator/coordination | Is there a compensation for care coordinator/coordination? | 4 experts (80%) | 1 expert (20%) |  |  |
|  | 10.5 Reimbursement for transitional care | Is there a reimbursement for transitional care? | 2 experts (40%) | 2 experts (40%) |  | 1 expert (20%) |
|  | 10.6 Out-of-pocket payments | Are out-of-pocket payments level low and do not affect patients & informal caregiver decision/possibility to access primary care? | 4 experts (80%) | 1 expert (20%) |  |  |
| **11. Hospital** | 11.1 Appropriateness of reimbursement level - sufficient reimbursement level to cover the costs? | Is the reimbursement level sufficient to cover the costs incurred? | 2 experts (40%) | 3 experts (60%) |  |  |
|  | 11.2 Presence of incentives that stimulate cost-efficient care | Are there any incentives (e.g. Pay for Performance, Pay for Quality etc.) in place to stimulate cost-efficient care? | 2 experts (40%) | 3 experts (60%) |  |  |
|  | 11.3 Sufficient renumeration level of the staff | Is the renumeration for staff sufficient to ensure the retention of staff? | 2 experts (40%) | 2 experts (40%) |  | 1 expert (20%) |
|  | 11.4 Compensation for care coordinator/coordination | Is there a compensation for care coordinator/coordination? | 3 experts (60%) | 2 experts (40%) |  |  |
|  | 11.5 Reimbursement for transitional care | Is there a reimbursement for transitional care? | 2 experts (40%) | 2 experts (40%) |  | 1 expert (20%) |
|  | 11.6 Out-of-pocket payments | Are out-of-pocket payments level low and do not affect patients & informal caregiver decision/possibility to access hospital? | 3 experts (60%) | 1 expert (20%) | 1 expert (20%) |  |
| **12. Long-term care** | 12.1 Appropriateness of reimbursement level - sufficient reimbursement level to cover the costs? | Is the reimbursement level sufficient to cover the costs incurred? | 2 experts (40%) | 2 experts (40%) | 1 expert (20%) |  |
|  | 12.2 Presence of incentives that stimulate cost-efficient care | Are there any incentives (e.g. Pay for Performance, Pay for Quality etc.) in place to stimulate cost-efficient care? | 2 experts (40%) | 2 experts (40%) | 1 expert (20%) |  |
|  | 12.3 Sufficient renumeration level of the staff | Is the renumeration for staff sufficient to ensure the retention of staff? | 2 experts (40%) | 1 expert (20%) | 1 expert (20%) | 1 expert (20%) |
|  | 12.4 Compensation for care coordinator/coordination | Is there a compensation for care coordinator/coordination? | 3 experts (60%) | 1 expert (20%) |  | 1 expert (20%) |
|  | 12.5 Reimbursement for transitional care | Is there a reimbursement for transitional care? | 2 experts (40%) | 2 experts (40%) | 1 expert (20%) |  |
|  | 12.6 Out-of-pocket payments | Are out-of-pocket payments level low and do not affect patients & c informal caregiver decision/possibility to access LTC? | 3 experts (60%) | 1 expert (20%) | 1 expert (20%) |  |
|  | 12.7 Financial contribution by social care institutions to cover LTC costs | Do social care institutions contribute to cover LTC costs for patients & informal caregiver that have financial problems to pay for LTC? | 3 experts (60%) | 1 expert (20%) | 1 expert (20%) |  |
